# Social capital and physical activity: a literature review up to March 2024

**DOI:** 10.3389/fpubh.2025.1467571

**Published:** 2025-02-12

**Authors:** Zhendong Gao, Chen Soon Chee, Roxana Dev Omar Dev, Yutong Liu, Jianhong Gao, Rui Li, Fangyi Li, Xiaoxiao Liu, Tao Wang

**Affiliations:** ^1^Department of Sports Studies, Faculty of Educational Studies, Universiti Putra Malaysia, Serdang, Selangor, Malaysia; ^2^Department of Sports Teaching and Research, Lanzhou University, Lanzhou, China

**Keywords:** social capital, physical activity, public health, social cohesion, social trust, social participation, norms of reciprocity, social networks

## Abstract

**Background:**

Social capital, as a multidimensional social science concept, plays a crucial role in promoting physical activity. Despite numerous studies exploring the relationship between social capital and physical activity, there is still a lack of systematic understanding of how different dimensions of social capital influence physical activity levels. This study aims to systematically review the literature up to 2024 on the relationship between social capital and physical activity, uncover the role of social capital in promoting physical activity, and identify its multidimensional impacts.

**Methods:**

We used a combination of search terms including “social capital” and “physical activity” to search the Web of Science, PubMed, Scopus, SportDiscus, and PsychINFO databases for English literature published up to March 1, 2024.

**Results:**

We identified 2,021 unique articles and reviewed 115 studies that met our inclusion criteria. These studies evaluated various dimensions of social capital, with key dimensions including social participation (34%), social networks (30%), social cohesion (30%), social trust (29%), overall social network (26%), social support (19%), safety (19%), norms of reciprocity (13%), social control (10%), satisfaction with the environment (8%), collective efficacy (4%), norms for physical activity (3%), and voting (1%). In studies exploring the relationship between social capital and physical activity, the majority of positive results in the hypothesized direction were observed in dimensions such as social cohesion, trust, participation, reciprocity, satisfaction with the environment, and overall social networks. In contrast, dimensions such as voting, collective efficacy, safety, control, and physical activity norms predominantly showed null or negative results. The results for social support were mixed, displaying positive, negative, and null outcomes, while findings for social networks were also predominantly mixed.

**Conclusion:**

This study reveals the significant role of social capital in promoting physical activity, particularly in the dimensions of social cohesion, social trust, social participation, norms of reciprocity, satisfaction with environment, and overall social network. When designing public health interventions in the future, it is crucial to tailor strategies to different populations and contexts to better leverage social capital in promoting physical activity.

## 1 Introduction

In the field of public health, understanding, and promoting physical activity has become a crucial issue (1–3), especially in the context of the global rise in chronic diseases and mental health problems (4–6). Physical activity not only reduces the risk of heart disease, diabetes, and certain cancers but also significantly enhances psychological well-being and overall quality of life (7–9). Despite the well-known benefits of physical activity, activity levels are influenced by various factors, including biological, environmental, psychological, and social factors (10–12). Among these factors, the role of social capital has increasingly garnered the attention of researchers as strategies to promote physical activity continue to be explored in the field of public health (13–15).

Social capital, as a multidimensional social science concept, has become a significant topic of research in public health, psychology, and sociology over the past few decades (16–18). Although the definition and measurement of social capital remain contentious, there is a scholarly consensus on its core elements, which include social networks, social participation, trust, reciprocity, and shared norms (19, 20). Generally, social capital is defined as the resources and advantages individuals or groups derive from their social networks, typically acquired through social interactions, trust relationships, and community participation (19, 21). The key dimensions of social capital involve personal attributes such as the quality and quantity of social networks, social support, and information channels, as well as collective attributes like the degree of mutual trust among community members and shared social norms and values (21, 22).

Social capital can be further subdivided into various operational types or dimensions, including structural and cognitive; bonding, bridging, and linking; strong and weak ties; and horizontal and vertical (20, 23, 24). Cognitive social capital can be understood as individuals' perceptions of interpersonal trust, sharing, and reciprocity (21). Structural social capital refers to the density of social networks or patterns of civic participation (21). Bonding social capital pertains to relationships within homogenous groups, such as those among family members, neighbors, close friends, and colleagues, also known as strong ties (24). Bridging social capital involves connections between individuals or groups across different power structures, such as those linking diverse racial and occupational backgrounds, referred to as weak ties (24). Linking social capital is considered the respect and trust relationships that exist among people interacting across formal or institutionalized power or authority gradients in society (24–26). Among these, bonding and bridging social capital are regarded as horizontal social capital, while linking social capital is seen as vertical social capital (24).

The importance of social capital lies in its inclusion of both individual-level interactions and relationships as well as group or community-level cooperation and cohesion (21). Previous studies have indicated that social capital is considered a protective health factor (27). Some research also suggests that high levels of social capital are associated with numerous positive health outcomes, such as lower mortality rates, better mental health, and reduced crime and violence (17, 20, 28). Although some researchers have pointed out potential “negative effects” of social capital on health outcomes (29) or found its effects to be insignificant (28), there remains substantial evidence linking social capital with self-rated health (22, 30).

In the context of the relationship between social capital and physical activity, qualitative studies have found that social capital is regarded as a key resource for initiating and maintaining physical activity (31). Research indicates that strong social networks and high levels of social participation at the individual level can encourage more active lifestyles, including regular physical activity (32–34). Community-level reciprocity and neighborhood trust norms are associated with higher levels of physical activity among urban adults (15). High social capital has been shown to be associated with regular Moderate-to-Vigorous Physical Activity (MVPA) in boys and with overall physical activity in girls (35). Additionally, social capital can promote physical activity through the dissemination of health information (14). However, it is important to note that the relationship between social capital and physical activity may vary across different populations, influenced by factors such as age, gender, socioeconomic status, and culture (36–38).

To our knowledge, most systematic reviews on the relationship between social capital and health have primarily focused on broad health outcomes and public health interventions (16, 17, 30), and there has not been a systematic review specifically addressing the relationship between social capital and physical activity. Furthermore, scholars have emphasized the need for future research to focus on the multidimensionality and multi-layered perspectives of social capital (16, 17). In light of this, the present study aims to systematically review relevant literature to deeply explore the heterogeneous evidence of the impact of the multiple dimensions of social capital on physical activity, thereby filling the current research gap. By identifying effective social capital-building strategies and their applicability across different social and cultural contexts, we hope to provide new insights for public health practice and policy-making to leverage social capital at the policy level to improve public health outcomes.

## 2 Methods

Based on previous systematic reviews on related topics (28, 30, 39), we conducted an English literature search in March 2024 using the Web of Science, PubMed, Scopus, SportDiscus, and PsychINFO databases, with a cut-off date of March 1, 2024. These databases were selected for their high credibility and wide recognition in the fields of public health and sports science. We used a combination of search terms including “social capital” and “physical activity.” The specific search strategy is detailed in Table 1. We excluded abstracts, conference proceedings, dissertations, book chapters, and articles published in non-peer-reviewed journals.

**Table 1:** Search strategy.

**Type of database**	**Searching type**	**Result**
Web of science	#1 KP =(“social capital” OR “social cohesion” OR “collective efficacy” OR “social trust” OR “social networks” OR “social participation” OR “social engagement” OR “social integration” OR “social relationships” OR “social ties” OR “reciprocity” OR “social connections” OR “social connectedness”) AND KP =(“physical activity” OR “motor activity” OR “physical exertion” OR “sports” OR “exercise” OR “leisure physical activities” OR “leisure activities” OR “physical exercise” OR “physical inactivity”) and Article (Document Types) and English (Languages) #2 TI= (“social capital” OR “social cohesion” OR “collective efficacy” OR “social trust” OR “social networks” OR “social participation” OR “social engagement” OR “social integration” OR “social relationships” OR “social ties” OR “reciprocity” OR “social connections” OR “social connectedness”) AND TI= (“physical activity” OR “motor activity” OR “physical exertion” OR “sports” OR “exercise” OR “leisure physical activities” OR “leisure activities” OR “physical exercise” OR “physical inactivity”) and Article (Document Types) and English (Languages) #3 #1 OR #2	620
Pubmed	(“social capital” [Title/Abstract] OR “social cohesion” [Title/Abstract] OR “collective efficacy” [Title/Abstract] OR “social trust” [Title/Abstract] OR “social networks” [Title/Abstract] OR “social engagement” [Title/Abstract]) AND “social participation” [Title/Abstract] OR “social integration” [Title/Abstract] OR “social relationships” [Title/Abstract] OR “social ties” [Title/Abstract] OR “reciprocity” [Title/Abstract] OR “social connections” [Title/Abstract] OR “social connectedness” [Title/Abstract]) AND (“physical activity” [Title/Abstract] OR “motor activity” [Title/Abstract] OR “physical exertion” [Title/Abstract] OR “sports” [Title/Abstract] OR “exercise” [Title/Abstract] OR “leisure physical activities” [Title/Abstract] OR “leisure activities” [Title/Abstract] OR “physical exercise” [Title/Abstract] OR “physical inactivity” [Title/Abstract])	187
Scopus	(TITLE-ABS-KEY (“social capital” OR “social cohesion” OR “collective efficacy” OR “social trust” OR “social networks” OR “social engagement” “social participation” OR “social integration” OR “social relationships” OR “social ties” OR “reciprocity” OR “social connections” OR “social connectedness”) AND TITLE-ABS-KEY (“physical activity” OR “motor activity” OR “physical exertion” OR “sports” OR “exercise” OR “leisure physical activities” OR “leisure activities” OR “physical exercise” OR “physical inactivity”))	439
SportDiscus	AB (“social capital” OR “social cohesion” OR “collective efficacy” OR “social trust” OR “social networks” OR “social participation” OR “social engagement” OR “social integration” OR “social relationships” OR “social ties” OR “reciprocity” OR “social connections” OR “social connectedness”) AND AB (“physical activity” OR “motor activity” OR “physical exertion” OR “sports” OR “exercise” OR “leisure physical activities” OR “leisure activities” OR “physical exercise” OR “physical inactivity”)	456
PsychINFO	title((“social capital” OR “social cohesion” OR “collective efficacy” OR “social trust” OR “social networks” OR “social participation” OR “social engagement” OR “social integration” OR “social relationships” OR “social ties” OR “reciprocity” OR “social connections” OR “social connectedness”)) AND title((“physical activity” OR “motor activity” OR “physical exertion” OR “sports” OR “exercise” OR “leisure physical activities” OR “leisure activities” OR “physical exercise” OR “physical inactivity”)) OR if((“social capital” OR “social cohesion” OR “collective efficacy” OR “social trust” OR “social networks” OR “social participation” OR “social engagement” OR “social integration” OR “social relationships” OR “social ties” OR “reciprocity” OR “social connections” OR “social connectedness”)) AND if((“physical activity” OR “motor activity” OR “physical exertion” OR “sports” OR “exercise” OR “leisure physical activities” OR “leisure activities” OR “physical exercise” OR “physical inactivity”))	319

The inclusion criteria were as follows: (1) Studies focusing on the relationship between social capital and physical activity, including observational studies (cross-sectional studies, prospective and retrospective cohort studies, case-control studies) and randomized controlled trials; (2) Studies that conducted formal hypothesis testing on the relationship between measures of social capital and physical activity; (3) Studies that measured physical activity using objective methods or subjective assessments, including but not limited to frequency, duration, and intensity of participation; (4) Studies that included at least one measure of social capital; (5) Studies published in peer-reviewed journals in English up to March 1, 2024.

The exclusion criteria were: (1) Studies that did not provide direct results on the association between measures of social capital and physical activity; (2) Reviews, opinion articles, or theoretical papers; (3) Studies where physical activity outcomes were indirectly obtained or measured through exercise behavior or exercise psychology; (4) Studies that were not available in full text or had incomplete data; (5) Studies that only included measures of social support. We excluded studies focusing solely on social support, as there are numerous reviews on this topic (39–41). We will focus on explaining social capital through the lens of social cohesion, as the search terms for social capital inevitably reveal methods based on social support and social cohesion (16, 30).

The search process involved a layered evaluation and adhered to the PRISMA guidelines (42) to ensure systematic and transparent literature screening. Initially, the identified literature was downloaded into Endnote X7 after removing duplicates, and studies were selected based on their titles and abstracts. Subsequently, the full texts of the remaining studies were retrieved and assessed for eligibility. If necessary, both abstracts and full texts were screened. The search and selection process was independently conducted by four researchers (ZG, YL, JG, and RL). Any discrepancies regarding the inclusion of specific studies were resolved through consensus meetings. If consensus could not be reached, the final decision on inclusion or exclusion was made by researchers (CC and RO). The basic information of each retrieved article (i.e., author, publication year, and article title) was recorded by the author (ZG) in a Microsoft Excel^®^ spreadsheet to ensure comprehensive tracking and review.

The data extraction process was carried out independently by five reviewers (ZG, YL, JG, RL, and FL) following standardized methods for systematic reviews. In cases of disagreement, the reviewers consulted researchers (CC and RO), and discrepancies were resolved through consensus. Key elements from each study were extracted and summarized in a table, organized chronologically by publication year. For each study, the table included the following: the first author and year of publication, sample information (size, characteristics, and location), study design (cross-sectional, prospective, or experimental), measures of social capital, measures of physical activity, covariates included as control variables, and main statistical results (effect estimates and/or significance of hypothesis testing). Bolded terms in the table indicated statistically significant results.

Additionally, we summarized the distribution of study outcomes, indicating whether the study authors reported “positive” results (significant associations in the hypothesized direction), “negative” results (negative and/or null associations), or “mixed” results (both positive and negative/null associations).

## 3 Results

### 3.1 Study selection

**Figure 1:**
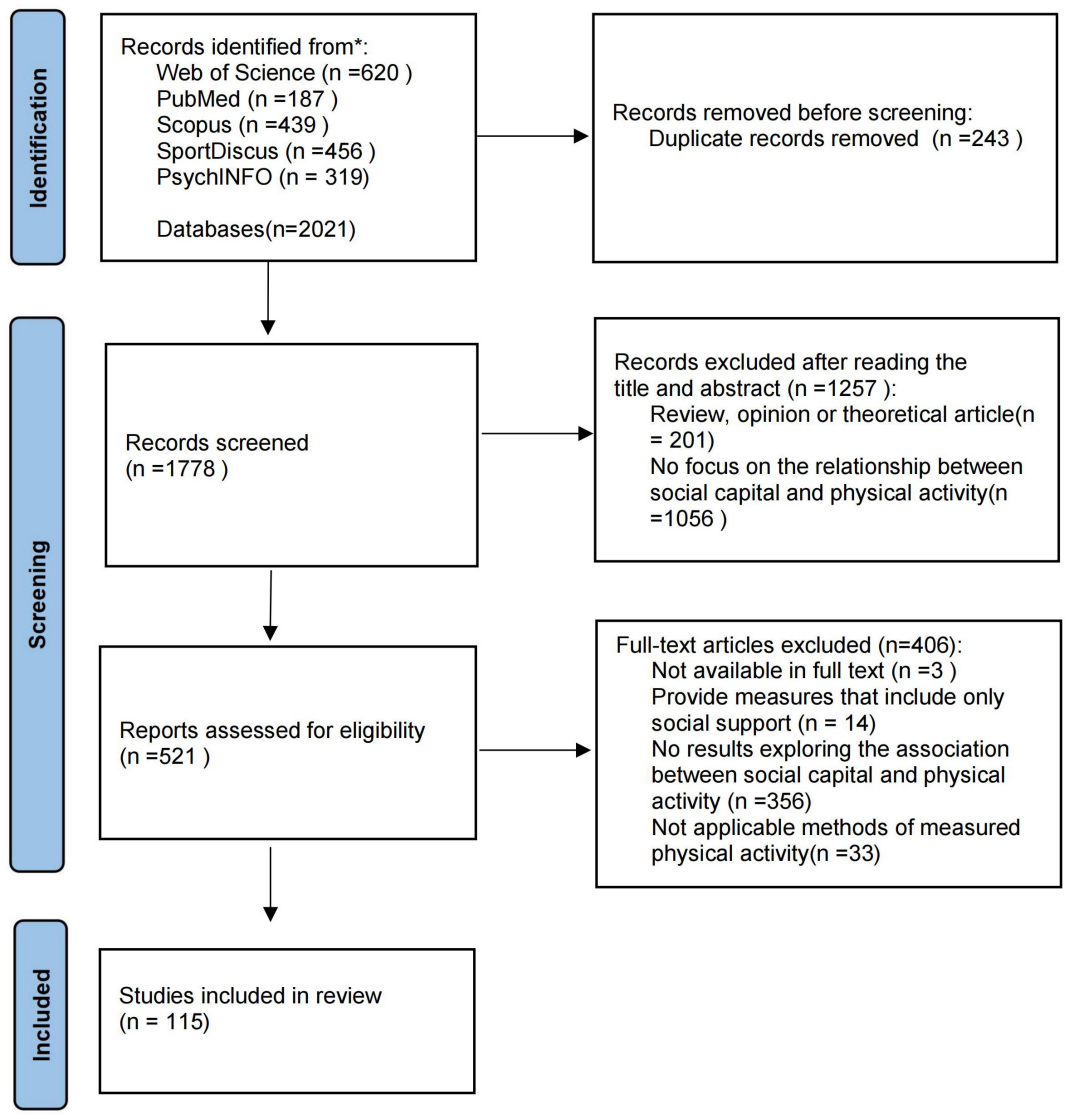
PRISMA flow diagram showing the process of study selection.

As shown in the PRISMA flow diagram [Figure 1, (42)], the initial search yielded 2,021 published papers. After removing duplicates, 1,778 papers remained. Upon reviewing titles and abstracts, 1,257 papers were further excluded because they did not focus on the relationship between social capital and physical activity (*n* = 1,056) or were reviews, opinion articles, or theoretical papers (*n* = 201). Of the remaining 521 papers, 406 were excluded after full-text review for the following reasons: providing measures that included only social support (*n* = 14), no results exploring the association between social capital and physical activity (*n* = 356), using non-applicable methods to measure physical activity (*n* = 33), and not being available in full text (*n* = 3). Consequently, 115 papers were included in this systematic review.

### 3.2 Study characteristics

This review summarizes the characteristics of the 115 included studies (see Supplementary Material 1 for details). Table 2 provides a summary of these characteristics. Among them, 89 (77%) studies used a cross-sectional design, and 26 (23%) used a longitudinal design. 53 (46%) studies involved samples from the United States, 10 (9%) involved samples from China, and 8 (7%) involved samples from Japan/Canada. Four (3%) studies each involved samples from Brazil/Sweden, and the remaining 28 studies involved samples from 24 different countries. The total sample size of the included studies reached 1,328,785, with 22 (19%) studies having a sample size of over 10,000 and 69 (60%) studies having a sample size of over 1,000. Seventeen (15%) studies used nationally representative data. The sample age range in the included studies was from 6 to 91 years. Twenty-four (21%) studies focused on samples of minors. The majority (79%) of the studies focused on adults, with 40 (44%) studies primarily involving middle-aged and older adults (50 years and above). Covariates controlled at the individual level included age (95%), gender (71%), education level (62%), marital status (30%), and race/ethnicity (27%). Ten percent of the studies included regional-level covariates (neighborhood, school, county). Additionally, 43% of the studies controlled for health-related covariates, and 24% controlled for socioeconomic status-related covariates.

**Table 2:** Summary of characteristics of the 115 included studies.

**Characteristic**	**Number**	**Proportion (%)**
**Study design**		
- Cross-sectional design	89	77%
- Longitudinal design	26	23%
**Sample sources by country**		
- The United States	53	46%
- China	10	9%
- Japan/Canada	8	7%
- Brazil/Sweden	4	3%
- Other 24 countries	28	24%
**Total sample size**	1,328,785	100%
Studies with large samples (>1,000)	69	60%
- Including samples >10,000	22	19%
Studies using nationally representative data	17	15%
**Sample age range (years)**	6–91	
Studies focused on minors	24	21%
Studies focused on adults	91	79%
- Among adults: middle-aged and older adults (50 years and above)	40	44%
**Controlled covariates**		
**-Individual level**		
- Age	109	95%
- Gender	81	71%
- Education level	71	62%
- Marital status	34	30%
- Race/Ethnicity	31	27%
- **Regional level** (Neighborhood, school, county)	11	10%
- **Health-related covariates**	49	43%
- **Socioeconomic status covariates**	28	24%

Table 3 summarizes the key attributes of the selected studies, including the distribution of social capital domains covered and the main research findings (positive, negative, and mixed). Among the 115 studies, the components measured across the studies varied significantly in frequency. The most commonly assessed components were social participation (34%), social networks (30%), and social cohesion (30%), which appeared in nearly one-third of the studies. In contrast, less frequently assessed components included collective efficacy (4%), norms for physical activity (3%), and voting (1%), highlighting their limited inclusion in the reviewed literature.

**Table 3:** Summary of social capital indicators included in the results of the social capital and physical health study (*n* = 115).

**Social capital indicator**	**Frequency (%)**	**Result**		
		**Positive**	**Mixed**	**Null/negative**
Social cohesion	34 (30%)	22	6	6
Trust	33 (29%)	16	5	12
Participation	39 (34%)	23	9	7
Reciprocity	15(13%)	7	4	4
Satisfied with environment	9 (8%)	5	1	3
Voting	1 (1%)			1
Collective Efficacy	5 (4%)	1		4
Safety	22 (19%)	1	5	16
Control	11 (10%)	4	1	6
Norms for PA	3 (3%)	1		2
Overall social network	30 (26%)	25	2	3
Social support	22 (19%)	7	6	9
Social networks	35 (30%)	5	26	4

Percentages may add to over 100% as many studies had measures from multiple social capital domains.

The studies varied in their use of social capital measures, including constructs (latent variables composed of indicators) and individual indicators (single items). 10% of the studies used a single measure of social capital (composed of one or more indicators), while 39% used a single social capital indicator only. 51% of the studies used multiple measures and indicators. The median number of indicators per study was 2, with a range of 1 to 6.

The most frequently examined measure of physical activity (PA) was subjective (86%), with 12 studies using objective PA measures. Four studies employed mixed measures. The types of PA included overall physical activity (79%), followed by leisure-time physical activity (17%), and 5 (4%) studies measured other types such as recreational, mixed, and school-based activities.

### 3.3 Overall findings of the 115 studies on social capital and physical activity

Among the 115 included studies, the majority of positive results in the hypothesized direction for the association between social capital and physical activity were found in the following indicators: social cohesion, trust, participation, reciprocity, satisfaction with environment, and overall social network (see Table 3). The indicators of voting, collective efficacy, safety, control, and norms for PA predominantly showed null/negative results. The results for social support were similarly distributed among positive, negative, and mixed outcomes, while social networks predominantly showed mixed results. It is worth noting that the included studies examined various aspects of social networks related to physical activity. Some studies focused on specific dimensions, such as family or peer networks, or particular aspects like network size, diversity, and tie strength; these were categorized as social networks. Other studies analyzed the overall characteristics of entire social network, which we classified as overall social network. This classification helps to clarify the variability in the study results, as studies focusing on specific network dimensions may capture more targeted social influences, while studies examining overall network reflect broader structural patterns.

### 3.4 Social cohesion and physical activity

A total of 34 studies examined the relationship between social cohesion and physical activity (38, 43–75). Overall, the studies found a direct positive correlation between social cohesion and physical activity. Specifically, 22 (64.7%) studies showed positive results, 6 (17.6%) studies showed mixed results, and 6 (17.6%) studies showed null or negative results.

The insignificant results were observed in specific subgroups: women/mothers (45, 49, 61), adolescents/high school students (55, 71), residents of high socioeconomic status/developed countries (53, 58, 66), middle-aged and older Chinese people (56, 70), and samples from low-income and socioeconomically disadvantaged populations (46, 60).

### 3.5 Social trust and physical activity

A total of 33 studies examined the relationship between social trust and physical activity (15, 35–37, 45, 49, 50, 53, 57, 61, 63, 67, 71, 75–93). Overall, the studies found a direct positive correlation between social trust and physical activity. Specifically, 16 (48.5%) studies showed positive results, 5 (15.2%) studies showed mixed results, and 12 (36.3%) studies showed null or negative results.

The insignificant results were observed in specific subgroups: adults in developed countries (53, 77, 78, 81, 83, 88), middle-aged people in developing countries (84), women/mothers/pregnant women (49, 61, 93), children/high school students (35, 71, 82, 85, 89, 90), and disadvantaged communities (57).

### 3.6 Social participation and physical activity

A total of 39 studies examined the relationship between social participation and physical activity (14, 37, 45, 46, 56, 63, 68, 71, 75–77, 79, 83, 84, 86–88, 91, 92, 94–113). Overall, studies found a direct positive correlation between social participation and physical activity. Specifically, 23 (59.0%) studies showed positive results, 9 (23.1%) studies showed mixed results, and 7 (17.9%) studies showed null or negative results.

The insignificant results were observed in specific subgroups: middle-aged and older adults (56, 104–107, 109, 111), adolescents (98, 101), adults in developed countries (77, 79), studies that could not predict longitudinal results (110), and some special samples such as social group members (68, 102), cancer survivors (99), and low-income adults living in public housing (46).

### 3.7 Social norms and physical activity

A total of 15 studies examined the relationship between norms of reciprocity and physical activity (15, 35, 46, 57, 67, 78, 80, 82–85, 91, 92, 114, 115). Overall, studies found a direct positive correlation between norms of reciprocity and physical activity. Specifically, 7 (46.6%) studies showed positive results, 4 (26.7%) studies showed mixed results, and 4 (26.7%) studies showed null or negative results.

The insignificant results were observed in specific subgroups: adults in developed countries (78, 83), minors (35, 82, 85), women (45), working-class populations (80), and disadvantaged community adults (57).

Three studies examined the relationship between norms for PA and physical activity (46, 68, 80). Among these, only one study targeting African American church members (68) reported positive results. The other two studies did not find significant results and involved low-income adults living in public housing (46) and working-class populations (80).

### 3.8 Satisfaction with environment and physical activity

A total of 9 studies examined the relationship between community satisfaction with the environment and physical activity (47, 50, 52, 56, 58, 74, 86, 96, 115). Overall, studies found a direct positive correlation between satisfaction with the environment and physical activity. Specifically, 5 (62.5%) studies showed positive results, 1 (11.1%) study showed mixed results, and 3 (37.5%) studies showed null or negative results.

The insignificant results were observed in specific subgroups: adolescents (47), older adults in China (56), and adults in developing countries (58). Adults in developed countries provided mixed results (52).

### 3.9 Social network and physical activity

A total of 30 studies examined the relationship between overall social network and physical activity (14, 15, 38, 45, 46, 53, 57, 60, 67, 77, 80, 83, 86, 87, 91, 92, 94, 97, 98, 106, 108, 116–124). Overall, studies found a direct positive correlation between overall social network and physical activity. Specifically, 25 (83.3%) studies showed positive results, 2 (6.7%) studies showed mixed results, and 3 (10%) studies showed null or negative results.

The insignificant results were observed in specific subgroups: women in developed countries (45), working-class populations (80), and male populations in developing countries (15). Mixed results were observed during the COVID-19 pandemic among older female populations and adults in developed countries (77, 123).

A total of 35 studies examined the relationship between social networks and physical activity (32, 34, 66, 81, 84, 88, 99, 102, 104, 105, 107, 112, 113, 125–145). Overall, the studies found that the relationship between social networks and physical activity was mixed. Specifically, 5 (14.3%) studies showed positive results, 26 (74.3%) studies showed mixed results, and 4 (11.4%) studies showed null or negative results.

The studies that showed only null or negative results were found in specific subgroups: cancer survivors (99), older college students with small sample sizes (135), Latino civic groups (102), and members of the same sociocultural organization (107).

### 3.10 Other indicators and physical activity

In studies examining the relationship between voting, collective efficacy, safety, and physical activity, the overall relationship was found to be null or negative. Specifically, only one study included voting and provided null/negative results (77). In studies examining collective efficacy (64, 89, 90, 146, 147), only one study reported positive results, which involved an intervention aimed at enhancing collective efficacy among mothers (146).

Regarding studies on safety (37, 43, 45, 47, 49, 51, 52, 58, 62, 73, 78, 80, 84, 85, 93, 105–107, 114, 127, 148, 149), 1 study (4.5%) showed positive results, 5 studies (22.8%) showed mixed results, and 16 studies (72.7%) showed null or negative results.

In studies examining the relationship between social control, social support, and physical activity, the overall relationship was found to be mixed. Regarding studies on social control (14, 35, 37, 60, 61, 71, 73, 89, 90, 108, 146), 4 (36.4%) studies showed positive results, 1 (9.1%) study showed mixed results, and 6 (54.5%) studies showed null or negative results. The insignificant results were observed in women (61), adolescents and children (35, 71, 73, 89, 90,), and socioeconomically disadvantaged populations (60).

In studies on social support (14, 32, 35, 46, 60, 61, 68, 71, 80, 82, 83, 86, 90, 93, 94, 97, 101, 108, 113, 127, 136, 138), 7 (31.8%) studies showed positive results, 6 (27.3%) studies showed mixed results, and 9 (40.9%) studies showed null or negative results.

## 4 Discussion

### 4.1 Summary of key findings

This study found that social capital plays an important role in promoting physical activity. Our analysis indicates that multiple dimensions of social capital, including social cohesion, social trust, social participation, norms of reciprocity, satisfaction with environment, and overall social network, are significantly associated with physical activity. However, dimensions such as voting, collective efficacy, safety, and norms for physical activity did not show a significant association with physical activity. Additionally, the relationships between social networks, social control, and social support with physical activity yielded mixed results. Due to the varying relationships between different dimensions of social capital and individual characteristics, most studies reported both positive and negative outcomes.

### 4.2 Positive outcomes

Social cohesion refers to the bonds and sense of solidarity among community members and is a crucial dimension of social capital (150, 151). Strong cohesion within a community can provide emotional support and increase opportunities for physical activity through community events and programs (152, 153). In this study, we found that social cohesion is positively associated with physical activity. This finding aligns with the existing literature and further underscores the critical role of social cohesion in promoting healthy behaviors (30, 154).

Trust is one of the core elements of social capital (155). High levels of trust within a community can enhance residents' sense of safety, indirectly promoting social support and cooperative social interactions, making individuals more willing to engage in outdoor activities and exercise (156–158). Overall, trust is positively associated with physical activity.

Social participation involves individuals' engagement in community activities and social organizations (159). Active participation in community activities not only increases opportunities for physical activity but also helps build more social connections, providing emotional support and social encouragement, thereby enhancing individuals' mental health (160–162). Overall, social participation is significantly associated with physical activity.

Norms of reciprocity refer to the mutual assistance and supportive behaviors among community members (163). In a community with strong norms of reciprocity, residents are more likely to help and encourage each other. This mutual support can provide both emotional support and practical assistance for physical activity (164, 165). Overall, there is a positive association between norms of reciprocity and physical activity.

Satisfaction with the environment refers to residents' overall attitude toward their community environment (166). Studies have shown that a good community environment, including factors like community density, green spaces, sports facilities, and street connectivity, can significantly enhance residents' levels of physical activity (167–169). Our results indicate that satisfaction with the environment is closely related to physical activity levels, with residents of highly satisfying communities being more likely to engage in outdoor activities and exercise.

Overall social network refers to the total sum of an individual's social relationships and connections, which can provide information, support, and motivation (170). In this study, we found that overall social network is significantly positively associated with physical activity. This finding aligns with the existing literature (171–173) and further emphasizes the critical role of overall social networks in promoting healthy behaviors (174, 175).

Although these social capital indicators generally have a positive impact on physical activity, their effects may vary depending on demographic characteristics and social context. For example, women showed insignificant results in indicators such as social cohesion, social trust, norms of reciprocity, and overall social networks. This could be because women, in many cultural contexts, bear more family and caregiving responsibilities, which limit their time and energy (176, 177). Social networks for women often focus more on family and close relationships, which may not directly promote physical activity (130, 178). Additionally, women might rely more on internal family support and trust rather than community-level trust to determine their physical activity behaviors (179).

Minors showed insignificant results in the social capital indicators of social cohesion, social trust, social participation, norms of reciprocity, and satisfaction with the environment. This may be because their physical activity is more influenced by school and family environments rather than the broader community (208). Additionally, some studies on social capital indicators for minors rely on parents' perceptions of family social capital (82, 85), which could affect the results. Parents' perceptions may not fully reflect the actual experiences and interactions of minors, leading to discrepancies in the findings.

Among adults, some insignificant results were observed in the social capital indicators of social cohesion, social trust, social participation, norms of reciprocity, satisfaction with the environment, and overall social networks, particularly among middle-aged and older adults, as well as adults in developed countries. This may be because the influence of social capital is confounded by other variables and external factors, indicating that its impact is not solely dependent on social capital itself (36, 180, 181).

Additionally, certain special populations, such as low-income and socioeconomically disadvantaged groups, specific social groups, and cancer survivors, as well as unpredictable longitudinal results, may reflect the protective potential of social capital. However, the unique challenges faced by these groups might limit its effects (182–184).

The type of social capital indicator can also create differences. For example, the frequency, type, and duration of social participation can influence its relationship with physical activity (102, 105, 106, 111). Moreover, the negative effects of social capital should not be overlooked, such as workplace social capital and social participation among students, which have shown negative associations with physical activity (80, 98).

### 4.3 Negative outcomes

Voting behavior is often considered a form of social participation (185). However, in this study, we found no significant association between studies involving voting behavior and physical activity. This may be because voting behavior itself does not directly involve physical activity, nor does it directly provide social support or enhance community interaction. Voting behavior mainly reflects citizens' willingness to participate politically rather than their daily health behaviors or physical activity levels (186). This could also be one of the reasons why there are fewer studies investigating the relationship between voting behavior and physical activity.

Collective efficacy refers to the ability of community members to work together to solve problems and achieve common goals (187). In this study, the association between collective efficacy and physical activity was not significant. This may be because collective efficacy more accurately reflects a community's ability to address social issues and provide public services rather than directly involving individual health behaviors (188, 189). Although communities with high collective efficacy may have better public resources and a safer environment (190, 191), this does not necessarily translate into individual physical activity behaviors.

Safety is often considered an important factor influencing physical activity (192, 193), but our study found no significant association between safety and physical activity. This is consistent with previous review findings (194). The lack of significant results may be due to varying measurement standards among the included studies, as broad crime indicators and PA measures might limit our ability to interpret the results. Additionally, while a safe community environment can provide the basic assurance for physical activity, merely feeling safe may not be sufficient to motivate individuals to increase their physical activity. Crime itself might not affect a person's PA behavior unless they feel threatened by crime or fear it (193).

Norms for physical activity refer to the community's expectations and promotion of physical activity (195). However, our study found no significant association between norms for physical activity and actual physical activity. This may be due to the limited number of studies involved, which focused on specific groups such as low-income adults (46) and working-class populations (80). Individuals' physical activity behavior may be more influenced by personal motivation, time management, and lifestyle (196, 197), rather than solely by community norms. Even if there are positive norms for physical activity within a community, the impact of these norms on individual behavior may be limited without the necessary facilities and support (198).

### 4.4 Mixed outcomes

Social networks refer to individuals' social relationships and connections, including family, friends, colleagues, and neighborhood ties. These networks can be measured by various characteristics, such as size, density, relationship quality, and composition (199). Our findings show that the relationship between social networks and physical activity is mixed. This is consistent with previous studies (200–202). The mixed results highlight that strong social networks can promote physical activity through multiple pathways (170). However, the effects of social networks may vary across different populations and social contexts, potentially leading to ineffectiveness in certain groups, such as cancer survivors (99), older college students with small sample sizes (135), Latino civic groups (102), and members of the same sociocultural organization (107).

Social control refers to the ability of community members to influence and regulate individual behaviors through formal or informal means (203). In the included studies, social control provided mixed results. However, insignificant results were observed in specific groups such as women (61), adolescents and children (35, 71, 73, 89, 90), and socioeconomically disadvantaged populations (60). This may stem from the dual nature of social control's impact on individual behavior, encompassing both positive and negative influences (204, 205). Its effects vary depending on the implementation method and community context.

Social support refers to the emotional, informational, and practical assistance individuals receive from others, and it is considered an important factor in promoting physical activity (206). Our results show mixed findings regarding the relationship between social support and physical activity. This is likely due to the limitations of our search strategy and inclusion criteria. Numerous existing reviews have already established the relationship between social support and physical activity (39–41).

### 4.5 Limitations and future directions

This study has several limitations. First, we only included literature published in English, which may have led to the exclusion of important studies in other languages. Second, most of the included studies used a cross-sectional design. While this design can reveal associations between variables, it cannot establish causality. Third, as a multidimensional concept, social capital has been measured using different methods and indicators across studies. This inconsistency can lead to heterogeneous results and limit the comparability of findings across studies. This is a common issue in social capital and health research, where the lack of consensus on the definition and measurement of social capital restricts researchers' ability to aggregate and quantitatively analyze results (30, 207). In our study, we combined unified social capital indicators with specific social capital indicators, which may have increased heterogeneity and potential confounding effects, thus limiting the robustness of our findings. Fourth, we adopted broad inclusion criteria for physical activity measures, and the studies primarily relied on subjective assessments of physical activity levels, which could result in reporting bias and measurement errors. Fifth, although the sample populations in the included studies are increasingly diverse, they are predominantly from developed countries, which limits the generalizability of the results and their applicability to different cultural contexts.

Based on the limitations discussed in this paper, future research should focus on: (1) adopting more longitudinal designs to better understand the long-term effects and causal mechanisms of social capital on physical activity; (2) striving for standardization and consistency in social capital measurement methods to improve the comparability of results and the ability to conduct comprehensive analyses; (3) using objective assessment tools to reduce potential biases and improve accuracy; (4) expanding the sample range to include more studies from developing countries and diverse cultural backgrounds to provide a more comprehensive understanding; (5) increasing focus on specific populations (such as minors, older adults, and socioeconomically disadvantaged groups) is needed, as there are fewer studies on these groups and the results are inconsistent. Future research should explore the role and impact of social capital in these populations in greater depth; (6) exploring the negative effects of social capital, as some studies have highlighted its potential adverse impacts. Future research should investigate these negative effects to fully understand their influence on physical activity and health; (7) developing and evaluating interventions aimed at promoting physical activity by enhancing social capital, and testing the underlying mechanisms and mediating effects, as most studies assume specific pathways but rarely test potential mechanisms.

## 5 Conclusion

This study systematically reviewed the literature on the relationship between social capital and physical activity up to 2024, revealing the significant role of social capital in promoting physical activity. Our review of studies meeting our inclusion criteria found substantial evidence of associations between multiple dimensions of social capital—such as social cohesion, social trust, social participation, norms of reciprocity, satisfaction with environment, and overall social network—and physical activity. Few studies found significant associations between physical activity and the dimensions of voting, collective efficacy, safety, and norms for physical activity. In contrast, the dimensions of social networks, social control, and social support showed more support for an association with physical activity, but the results were mixed. Most studies yielded both positive and null results. These findings highlight the protective role of social capital in physical activity, indicating a strong influence of individual characteristics and cultural backgrounds. In the future, public health interventions should be tailored to different populations and contexts to better leverage social capital in promoting physical activity.
