# Supplementary material for: Social capital and physical activity: a literature review up to March 2024
Source: Front Public Health. 2025 Feb 12;13:1467571. doi: 10.3389/fpubh.2025.1467571 (PMC11860974; doi:10.3389/fpubh.2025.1467571)
Supplement: Supplementary file 1 [file Table_1.docx]

Supplementary Material

| Results of literature review | | | | | | | | |
| --- | --- | --- | --- | --- | --- | --- | --- | --- |
| Title | Study Design | Participant characteristics (age [mean and range], gender) | Location | Cultural background | Social capital indicator | Physical activity indicators | Covariates | Result |
| (Unger & Johnson, 1995) | Cross-sectional | N=200 (Mage=39.3, SD = 10.5; AR=21 ~ 79) | southern California, America | Mostly Caucasian and well-educated, | 3-question social relationships (Social network) | Exercise frequency | *Individual level:* age, gender, and marital status | **Social network:** F=11，β>0,p < 0.05; |
| (Lindström et al., 2001) | Cross-sectional | N=11,837 (AR<65) | Malmo, Sweden | Na | Social network; social support; social participation | Low leisure-time physical activity | *Individual level:* age, country of origin and previous/current self-reported  diseases | **Low social network:** M: OR= 1.5 (1.3–1.7), F: OR =1.3 (1.2–1.5);  **low social support**: M: OR= 1.1 (1.0–1.3), F: OR =1.2 (1.1–1.4);  **low social participation**: M: OR =2.2 (2.0–2.5), F: OR =2.3 (2.0–2.6) |
| (Lindström et al., 2003) | Cross-sectional | N=3377(AR=20-80) | Malmo, Sweden | Na | 13 items social participation | Low leisure-time physical activity | *Individual level:* age, sex, country of origin, education | **low social participation:** OR =3.59 (2.95-4.35) |
| (Fisher et al., 2004) | Cross-sectional | N=582 (Mage= 73.9，SD=6.25 AR>65) | Oregon, America | Mostly Caucasian | 5 items social cohesion; neighborhood problems(violence), safety for walking activity in the neighborhood（crime） | Neighborhood walking activity (leisure) | *Neighborhood level:* low income, senior population density, percentage of White, and facilities per neighborhood | **Social Cohesion:** *neighborhood level***,** B= 0.034, t = 2.033, p < 0.05; Violence & Crime**：***Not significant* |
| (Greiner et al., 2004) | Cross-sectional | N=4601 (nationally survey) | Kansas, America | Mostly Caucasian and high proportion of rural residents | Trust and social participation | Physical activity | *Neighborhood level:* population density | **Trust:** OR =1.22 (1.13-1.32) p< 0.001 **social participation:** OR =1.62 (1.31-2.01) p < .001 |
| (Voorhees et al., 2005) | Cross-sectional | N=488 (Adolescent girls) | America | Na | Social networks | Physical activity | Na | **Social networks:** Univariate analysis ：most indicators significantly correlated  multivariate analysis：Frequency of PA with friends significantly correlated |
| (Zlot et al., 2006) | Cross-sectional | N = 2181 (nationally survey) | America | Na | Social capital barriers (Social participation ；satisfaction with government) | Leisure &  transport-related physical activity (not meeting physical activity recommendations) | *Individual level:* age, sex, marital status, education, race, household income, *Neighborhood level:* geographic region in the US, and population density | **Social capital barriers：**medium vs. low OR=1.56, [1.20-2.04] ~ 1.61, [1.18-2.22];  high vs. low OR=2.08 [1.56-2.78] ~2.12 [1.51-3.03] |
| (Kim et al., 2006) | Cross-sectional | Two-Level Analyses  N=167,857 adult; Three-Level Analyses  N = 94,145 adult (nationally survey) | America | Adults in developed countries | 14 items from Putnam State-level social capital index (ST1: Social network, engagement, trust; ST2: Voting & engagement); 6 items County-level:(CTY1: Social engagement, volunteering; CTY2: social network, trust | Lack leisure-time moderate to vigorous physical activity | *Individual level:* age, gender, race/ethnicity, marital, status education level, household income;  *County level:* Gini coefficient for 1995  , state-/county-level mean household income, percentage of black residents, urban sprawl | *State-level* social capital**：** 'ST1’: OR=0.91 [0.78–1.06] *Not significant*  'ST2’: OR=0.97 [0.83–1.13] (Not significant)  Combined: OR=0.86 [0.73–1.02] *Not significant*  *County-level* social capital**：**  '**CTY1**’: OR=0.94 [0.89–0.99]  'CTY2’: OR=0.98 [0.93–1.04] *Not significant*  **Combined**：OR=0.91 [0.86–0.97] |
| (Wen et al., 2007) | Cross-sectional | N=907 (Mage= 43.92, SD=15.69 | Chicago, America | Adults in developed countries | Neighborhood social capital (trust, norms of reciprocity, historical crime rates) | Leisure-time physical activity (regular exercise) | *Individual level:* age, gender, race/ethnicity, marital status, education, and annual household income | Neighborhood Trust**:** OR= 1.39 (p < 0.10)  Norms of Reciprocity**:** OR= 1.45 (p < 0.10)  **historical crime rates** (Homicide Rate:: 1991–93)**：**OR= 0.79 (p < 0.05)  Social Capital Index:  OR=1.20 (p < 0.05)  ****gender*** OR=1.37 (p < 0.05)  *gender & deprivation OR=1.10 (p < 0.05) |
| (Mummery et al., 2008) | Cross-sectional | N=1150 adults (Mage=74) | Queensland, Australia | Na | 9 items from British General Household Survey (GHS) Social Capital questions (social support, social participation, social network) | Physical inactivity | *Individual level:* Age, gender, years of education, and annual household income | **Social Capital**: Third quartile OR =0.43 [0.28-0.66], p < 0.05.  Highest quartile OR = 0.33 [0.21-0.52], p < 0.05 |
| (Azar et al., 2009) | Prospective | N=106, after 12months N=64  (Single parents) | Victoria, Australia | Na | Social network | Physical activity | Na | **Social network:** Z = −2.15, p = 0.032 (p < 0.05).  But increased Perception of Activity |
| (Cradock et al., 2009) | Longitudinal cohort | N=680 adults (AR= 11-15) | Chicago, America | Na | Neighborhood social cohesion | Physical activity | *Individual-Level:* age, sex, overweight status, race/ethnicity, household education level.  *Neighborhood-Level:* Availability of youth services and neighborhood educational attainment | **Neighborhood social cohesion:**  *Follow-up* β= 1.182, (p < 0.001). |
| (Ball et al., 2010) | Cross-sectional | N=1450 female (AR= 18-65) | Melbourne, Australia | Women | Social participation, social network;  *community-* interpersonal trust, norms of reciprocity, social cohesion, crime | Leisure-time physical activity | *Individual-Level:* age, education level, marital status, presence of children in the home, current pregnancy, and length of residence in the neighborhood | **social participation:** OR =3.30 [2.15-5.05], p < 0.05.  social network**:** OR =1.29 [0.75-2.23], *Not significant*  **interpersonal trust:** OR =1.73 [1.01-2.98], p < 0.05.  norms of reciprocity: OR =0.81 [0.47-1.39], *Not significant*  social cohesion**:** OR =1.71 [0.96-3.05], *Not significant*.  **crime:** *Not significant* |
| (Ueshima et al., 2010) | Cross-sectional | N=2260 (Mage=52.9±16.6, AR=20-80) | Okayama, Japan | Na | Cognitive Social Capital：trust  Structural Social Capital: Participation into bonding and bridging | Physical inactivity | *Individual-Level:* age, sex, educational attainment, family structure, self-rated health, mental status, and BMI. | **Trust:** OR = 0.58， [0.42–0.79] , p < 0.001.  Participation:  Bridging, OR = 0.79, [0.62–1.00], *Not significant*  bonding, OR = 0.71, [0.48 1.03]*, Not significant* |
| (Peláez et al., 2010) | Cross-sectional | N=756 cardiac outpatients. (Mage=60, AR=27-84) | Québec, Canada. | Na | Social networks | leisure-time physical activity (transformed MET) | *Individual-Level: age*, sex, previous major cardiovascular disease events, and fitness level | **Social networks**  relations network: F = 0.65, P = 0.422 *Not significant*  Source: F = 6.98, P = 0.008  Size: *Not significant* |
| (Shelton et al., 2011) | Prospective | N=1112 | Massachusetts, America | Low-Income Adults Living in Public Housing | Social network, social participation, social support, social cohesion, social norms for PA | Physical activity (measured with pedometers) | *Individual-Level:* employment status, gender, poverty level, current health status, age, perceived safety | *Just adjust age*: **social network** (P<0.001), **social cohesion** (P = 0.01)  *after adjust:*  **Social network:** 1503.7 (SE = 510.9), P =0.01 participation: *Not significant*  social support: *Not significant*  social cohesion**:** P = 0.26；b = 171.4, SE = 159.1 *Not significant*  social norms: *Not significant* |
| (Utter et al., 2011) | Cross-sectional | N=9107 (AR=13-18) (nationally survey) | New Zealand | Na | Neighborhood cohesion, safety, disintegration(satisfaction) | Physical activity | *Individual-Level:* age, gender, ethnicity, and deprivation index. | **Cohesion:** B = 0.081, SE = 0.036, P = 0.025  safety**:** p=0.599, *Not significant* disintegration(satisfaction): p=0.105, *Not significant* |
| (Lindström, 2011) | Cross-sectional | N=27,757 (AR=18-80) | Skåne County, Sweden | Na | Low trust | Low leisure time physical activity | *Individual-Level:* Age, country of origin, education, and desire to increase physical activity | **Trust:**  M: OR = 1.3 [1.1-1.4]  F: OR = 1.2 [1.1-1.3] |
| (Yu et al., 2011) | Cross-sectional | N=4107 (AR>16) | London, United Kingdom | Participants from disadvantaged areas | Social networks, support, crime | Leisure time physical activity (transformed MET) | *Individual-Level:* age, gender, education, job status, personal income, and perceptions of neighborhood safety | **Social networks:** individual-level predictors, Relatives: *Not significan*t  Friend: IRR = 1.02，[ 0.998 - 1.037] ,*Not significant*  Neighbor: IRR = 1.06，[1.023 - 1.101]  Social Support: *Not significant*  individual and area -level predictors Crime**:** IRR=0.70 [0.663- 1.306]  Social networks& Support: *Not significant* |
| (McNeill et al., 2012) | Prospective | N=850 adults (Mage=44) | London, United Kingdom | Working-class populations | Social capital (support, reciprocity, trust), norms for PA, safe, social network | Leisure-Time Physical Activity (on change in hours of leisure-time physical activity) | Na | **Social Capital** (support, reciprocity, trust):β=-0.80，p=0.01  social network**:**β=0.36，p=0.08 *Not significant*  safety & norms *Not significant* |
| (Shiovitz-Ezra & Litwin, 2012) | Cross-sectional | N=1716 (Mage=54.8±16.4，AR=57-85) (nationally sample) | America | National sample of older adults in the America | Social networks (diverse, friends, congregant, family, restricted) | Physical activity | *Individual-Level:* age, gender, income, race/ethnicity, self-rated health, activities of daily living difficulties | **Social networks**  **Friends:** *as Reference categories* OR=1.57, p<0.01  Diverse: OR = 0.91, [0.61–1.39], *Not significant*  **Family:** OR = 0.64, [0.42–0.98], p <0.05  Restricted: OR = 0.77, [0.52–1.13], *Not significant*  Congregant: OR = 0.80, [0.52–1.23], *Not significant* |
| (Legh-Jones & Moore, 2012) | Cross-sectional | N=2707 (AR≥25) | Montreal, Canada | Adults in developed countries | Social networks, social participation, trust | Physical inactivity (transformed MET) | *Individual-Level:* Gender, age, household income, educational attainment, and self-reported health | **Social networks:** Diversity OR=0.87, [0.80-0.95], Reach & Range: *Not significant*  **No social participation:** OR=1.64, [1.06-2.54]  Low trust**:** OR=1.07, [0.85, 1.36], *Not significant* |
| (Gesell et al., 2012) | Prospective | N=81 children（Mage=7.96） | Tennessee, America | Na | Social networks (number, category) | Physical activity (using accelerometers MVPA) | Individual-Level: age, obesity status, gender | **Social networks:** *category* OR = 6.89 (P <0.01)  *number: Not significant*  (versus making no change) |
| (Kaczynski & Glover, 2012) | Cross-sectional | N=380 adults | Ontario, Canada | Na | Social connectedness (trust, social cohesion) | Physical activity (measured in minutes) *for* *recreation or transportation* | *Individual-Level:* age, gender, body mass index (BMI), education level, and neighborhood of residence | **Social connectedness:** *for recreation* F = 11.36，P < 0.01  *transportation* F = 8.12，P < 0.01） |
| (Davison et al., 2012) | Cross-sectional | N=767 families (Children AR=6-19) | New York counties, America | Rural, predominantly white participants | Parent-perceived individual level family’s social capital (trust, reciprocity), social support | Physical activity (parents’ reports 60 min of PA per day) | *Individual-Level:* child age, household education, participation in food assistance programs, perceived neighborhood safety | for Younger Children (ages 6-12) **social capital：**sample model：β= 0.135 p<0.05; full model： *Not significant*  **social support:** *full model* β =0.223, p<0.001  Among older children（13-19）：**social capital：**sample model：β = 0.211 p<0.001; full model：β = 0.128 p<0.05  **social support:** *full model* β =0.279, p<0.001 |
| (Logstein et al., 2013) | Cross-sectional | N=8114 adolescents (Mage=16±1.72, AR=13-19) | Nord-Trøndelag, Norway. | Na | Informal social participation (social network), cultural participation (social participation) | Physical activity (exercise time outside school every week) | *Individual-Level:* age, sex, family economic affluence;  *community level:* social and economic deprivation indicators | Informal social participation **(social network):** β=0.1, p<0.01  cultural participation (social participation): β=-0.088,*Not significant* |
| (Nieminen et al., 2013) | Cross-sectional | N=8028 (AR≥30) (nationally survey) | Finland | Na | Social support, social participation and network, trust and reciprocity | Leisure-time physical activity | *Individual-Level:* gender, age group, education, living arrangements, household income. | Social support**:** OR = 1.04, [0.89-1.23], *Not significant*  **social participation and network:** OR = 4.73 [3.97-5.62], P<0.001  Trust and reciprocity**:** OR = 1.02, [0.87-1.20] *Not significant* |
| (Strong et al., 2013) | Cross-sectional | N=1374,(Mage=45±12.9) | Texas, America | African American community | Social cohesion and trust, traffic and road safety problems (safety) | Physical activity | *Individual-Level:* gender, age, education, income, employment status, marital status, presence of children, and years living in the neighborhood. | **Social cohesion and trust:** F: OR=1.06, [1.02, 1.11], p=0.006;  M: OR=1.07 [0.98, 1.17] p=0.141 *Not significant.*  **Safety:** F: OR=0.90, [0.64, 1.27], *Not significant*;  M: OR=0.37 [0.18, 0.73] p<0.01. |
| (Button et al., 2013) | Cross-sectional | N=18,875 (grades 6–10) (national survey) | Canada | Canadian school-age population | School social capital (trust, cohesion, Satisfied) | Physical activity at school (MVPA) | *Individual-Level:* socioeconomic status, grade, gender; *School-level:* urban-rural school location, school size. | **School social capita**: β=0.074, P=0.001. |
| (Larsen et al., 2014) | Cross-sectional | N=33,326 adults (national survey) | America | Na | Social integration (social networks) | Physical activity (MVPA) | *Individual-Level:* Age, gender, education, race/ethnicity, marital status. | Social integration (social networks): OR = 1.19, p ≤0.001;  Friend: OR = 1.93, p ≤ 0.001;  Family: OR = 1.21, p ≤ 0.01; |
| (Marquez et al., 2014) | Cross-sectional | N=393 (Mage=43.4, AR=18-89) | California, America | Predominantly Latino networks, family-based, speaking Spanish. | Social networks (size and composition) | Leisure-time physical activity (Meeting recommendation for LTPA) | *Individual-Level:* gender, age, education, income, marital status, health insurance, nativity, language proficiency. | **Social networks:** OR = 2.30, p=0.04. |
| (Pabayo, Molnar, et al., 2014) | Cross-sectional | N=1364 adolescents (AR= 14-19) | Massachusetts, America | Urban adolescents | Neighborhood & Individual social cohesion and safety | Physical inactivity | *Individual-Level:* age, gender, nativity, nativity race/ethnicity;  *Neighborhood-Level:* economic deprivation, social fragmentation, neighborhood disorder | Neighborhood social cohesion**:** OR=1.26[0.91，1.74], *Not significant.*  Safety**:** OR= 1.00[0.74，1.35], *Not significant.*  Individual social cohesion **:** OR=0.73[0.46,1.18], *Not significant.*  safety: R=0.92[0.59,1.44], *Not significant.* |
| (Jongeneel-Grimen et al., 2014) | Longitudinal | N1_2006_=25,309,  N2_2009_= 31,783  (AR= 18-84) (national survey) | Netherlands | Na | Neighborhood-level social cohesion, fear of crime, satisfied with environment(green space， Physical disorder，parking facilities) | Physical activity (hours per week) | *Individual-Level:* gender, age, employment status, education level, household income, and urbanization degree | **Neighborhood-level social cohesion:**  *In 2006* OR=1.16,[1.11，1.22],p≤0.05;  *Change between 2006 and 2009* OR=1.12,[1.05，1.19], p≤0.05  No fear of crime  *In 2006* OR=1.38,[1.28，1.51],p≤0.05;  Change between 2006 and 2009 OR=1.10,[0.99，1.22], *Not significant*  *satisfied with environment provides mixed results* |
| (Moore et al., 2014) | Cross-sectional | N=2616 (AR= 25-65 & above) | Montreal, Canada | City dwellers in developed countries | Social network, trust, cohesion | Physical inactivity (transformed MET) | *Individual-Level:* age, gender, socioeconomic status, educational background, employment status | **Social network:** β=-0.03, [-0.05, -0.01], p<0.05  trust: β=-0.01, [-0.04,0.02], *Not significant.*  cohesion: β=-0.01, [-0.03,0.01], *Not significant.* |
| (Prins et al., 2014) | Cross-sectional | N=832 adolescents (Mage= 13) | Rotterdam, Netherlands | Na | Neighborhood social capital (cohesion) | Physical activity (participating in sports at least three times per week) | *Individual-Level:* age, gender, ethnic;  *neighborhood-Level:* school level, neighborhood wealth, urbanization | **Neighborhood social capital** (cohesion): OR=5.40,[1.13，25.74], p<0.05; |
| (Pabayo, Janosz, et al., 2014) | Longitudinal, cohort | N=14924 students (AR= 12-18) | Quebec, Canada | Na | School Level social cohesion | Physical inactivity | *Individual-Level:* age, gender, immigration status family status, familial adversity | **Social cohesion:** OR=0.70,[0.58，0.85] |
| (Shin et al., 2014) | Longitudinal, cohort | N= 557 children (grade_5th_: Mage=10.74; grade_6th_: Mage=11.58) | Southern California, America | Na | Peer social networks | Physical activity (at school& out of school) | *Individual-Level:* age, gender, ethnic background, socioeconomic status, baseline behaviors, total nominations made (out-degree) | **Peer social networks**:  Peer exposure (sum)  at school b = 0.74，p<0.001  Outside of school b = -0.14, *Not significant*  Out-degree  at school b = -0.16，p<0.001  Outside of school b = 0.09, *Not significant* |
| Kim et al. (2015) | Cross-sectional | N=873, (Mage=64.5)  A nationally representative sample. | America | Cancer survivors | Social networks (marital status,network strength  ), social participation(  community organization membership) | Physical activity (≥150 min/week of moderate-intensity physical activity) | *Individual-Level:*  gender,age,body mass index (BMI),ethnicity,education income, insurance status, family history of cancer, health status | Social networks  *marital status* OR=1.47 (0.96, 2.24) *Not significant*  *network strength* OR=1.47 (0.93, 2.33) *Not significant*  *social participation* OR=1.07 (0.63, 1.78) *Not significant* |
| Gao et al. (2015) | Cross-sectional | N=2783, (AR≥60) | Shanghai, china | Older adults in a rapidly urbanizing chinese society | Social participation,  social cohesion, aesthetic quality(satisfied with environment) | Leisure-time physical activity | *Individual-Level:*  sex,age,marital status, self-reported chronic diseases,education self-rated health | *Individual level*  **Social participation** *quartile2th~4**th* OR=1.86~4.27 (1.44–5.58),  **social cohesion** *quartile2th~4th* OR=1.09~1.31 (1.07–3.50)  aesthetic quality *Not significant*  *Neighborhood level*  *all Not significant* |
| (Ruijsbroek et al., 2015) | Longitudinal | N=47926,(AR≥18)  2009N=192,015, 2011N =216,840;  National surveys | Dutch | Na | Social safety(objective,subjective) | Physical inactivity | *Individual-Level:*  age, sex, ethnicity, household composition, educational level, household income, urbanicity, crime frequency/ unsafety feelings ,  neighbourhood poor self-rated health | **Feeling unsafe at home** *High increase*  OR=1.12 (1.00–1.26)  others *all* *Not significant* |
| (Marlier et al., 2015) | Cross-sectional | N=414,(AR=18-56) | Antwerp, belgium | Disadvantaged communities | community social capital(social cohesion,trust,social network, reciprocity), individual social capital(trust,reciprocity) | Total physical activity | *Individual-Level:*  age, gender, education, ethnicity, tenancy, civil status,income, unemployment rate, ethnicity, population density. | **Community social capital**  β = 0.114, p < 0.05  individual social capital  β = -0.013, p > 0.05 *Not significant* |
| (Loch et al., 2015) | Cross-sectional | N=1062,(AR=40-90) | Cambé paraná brazil | Middle-aged people in developing countries | Social networks,reciprocity,trust,participation,safety | Insufficient leisure-time physical activity | *Individual-Level:*  gender, age group, socioeconomic condition, education, body mass index, mobility limitation, reported chronic condition, self-perceived health | Social networks  ***size*** OR=2.45 (1.26-4.77)，*strength* OR=1.03 (0.71-1.50) *Not significant*;  **reciprocity** OR=1.46 (1.06-2.02)  trust OR=1.17 (0.84-1.62) *Not significant*  **participation** OR=1.47 (1.05-2.06)  safety OR=1.31 (0.90-1.91), *Not significant*  **Social capital score**1.70 (1.07-2.70),p=0.002 |
| (Kennedy-Hendricks et al., 2015) | Cross-sectional | N= 209 children and their caretakers  (Mage=13±0.2,AR=8-18) | Montgomery County, America | The majority of the children lived in single-parent households, primarily with single mothers. | Caretaker social networks | Children's physical activity (at least 20 minutes every day) | *Individual-Level:* age, gender, race/ethnicity, the health composition of the child’s own social network,the head of household’s nativity, gender, educational attainment, and social network density; the number of years the family has lived in the neighborhood, household income-to-poverty ratio, family structure,caretaker’s corresponding health behavior or health characteristic | **Caretaker social networks**  OR=1.34,(1.07,1.69),  p <0.05. |
| (Johnson-Lawrence et al., 2015) | Cross-sectional | N=696 adults (AR≥25) | Detroit, michigan,  America | Non-hispanic black, non-hispanic white, and latino adults aged 25 or older | Social participation | physical activity (walking and moderate-intensity activities) | *Individual-Level:* age, gender, education level, poverty-to-income ratio, labor force participation, car ownership, home ownership status, marital status, physical limitations, race/ethnicity.  *Neighborhood-Level:* residential density , neighborhood poverty | **Social participation**  β= 0.17,SE = 0.09,p = 0.046 |
| (Andrade et al., 2015) | Cross-sectional | N=3597  (Mage=41.1±16.2,AR≥18) | Belo horizonte, minas gerais, brazil | Residents of Belo Horizonte with varying socioeconomic | Social cohesion, safety, services & aesthetics (satisfied with environment) | Leisure-time physical activity | *Individual-Level:*  age, sex, marital status, employment status, duration of residence, educational attainment, family income, regular fruit and vegetable consumption categorized ,smoking status, alcohol consumption, self-perception of health,social participation,  physical activity social support | **Social cohesion**  *Low Socioeconomic status*  OR = 1.43,(1.02-2.01）  others *Not significant*  safety, services & aesthetics all *Not significant* |
| (Muthuri et al., 2016) | Cross-sectional | N=563, (AR=9-11 ) | Nairobi, Kenya | Children | *Parental Perceptions* neighborhood social cohesion(reciprocity,trust),safety | Physical activity (using accelerometers MVPA & self-reported sufficient activity) | Na | *univariate analysis*  using accelerometers MVPA  **trust**: p = 0.048  reciprocity: p = 0.1148 *Not significant*  self-reported sufficient activity all *Not significant*  *Multivariable modeling*  all *Not significant* |
| (Babey et al., 2016) | Cross-sectional | N=2799, (Mage=14.6± 0.05,AR=12-17 ) | California, America | Adolescent /low-income youth and youth of colors | Social participation, (volunteer, organizations)school support | Physical activity (not including school PE,) | *Individual level:* age, gender, race/ethnicity, household income,region of residence | *Overall model*  **Social participation** *organizations*  β=0.38, p <0 .05  *Volunteer*  β=0.29, p <0 .05  **school support**  β=0.07, p <0 .01;  In the layered model, mixed results are provided. |
| (Calogiuri, 2016) | Cross-sectional | N=2168,(AR≥18  ) | Norway | Na | Social networks for NE-based PA (Social networks) | Physical activity (minutes per week) | *Individual level:*gender, age,educational level,  yearly household income，  co-inhabitation nucleus,  responsibility for small children,  region of residence  Centrality of residence,  objective measure of NE coverage within the municipality | **Social networks**  *Cut-Off 60 min/Week*  OR=1.88，(1.59–2.23)，p< 0.001  Cut-Off 150 min/Week  OR=1.67，(1.37–2.05)，p< 0.001 |
| (Harmon et al., 2016) | Cross-sectional | N=40,(Mage=25.4± 7.9 , AR≥18  ) | Hawaii, America | College student | Social networks (network nominations) | Meets recommendations MVPA (≥30 min/day) | *Individual level:* gender, age, ethnicity, screen time, percentage of fat intake, daily fruit and vegetable intake | Social networks OR=1.09 (0.90-1.31), *Not significant* |
| (Novak et al., 2016) | Cross-sectional | N=3428,(AR=17~18) | Zagreb City, Croatia | high school students | Family social capital (social support), neighborhood social capital(neighborhood trust，informal social control), school social capital (vertical & horizontal  school trust, reciprocity at school) | Overall physical activity & moderate-to-vigorous physical activity (MVPA) | *Individual level:* self-perceived socio-economic status,self-rated health,psychological distress,nutritional status(BMI) | *For MVPA*  **Boys High family social capital** OR=1.49 (1.18–1.90),p< 0.001  **Boys High informal social control** OR=1.26 (1.02–1.56),P< 0.05  *For overall physical activity*  **Girls High informal social control** OR=1.38 (1.09–1.76)，P < 0.01  **Boys High vertical school trust** OR=0.75 (0.57–0.98),P< 0.05  others *not significant* |
| (Becky et al., 2016) | Cross-sectional | N=335,(Mage=42± 16.4 , AR≥18  ) | Southern San Diego County, America | Latino Civic Group | Civic group participation, social network size (social networks) | Physical activity (MVPA) | *Individual level:* sex,age,marital status,education,  employment status,  acculturation | **Health group participation** β=0.915 , P=0.011；Religion, health, neighborhood and the arts *not significant*  social network size *not significant* |
| (Yi et al., 2016) | Cross-sectional | N=64754,(Mage=60.87, AR=18~65),nationally representative data | America | Non-Hispanic white, Non-Hispanic black, Hispanic, Non-Hispanic Chinese, Non-Hispanic Filipino, Non-Hispanic Asian Indian | Neighborhood social cohesion | leisure-time physical activity(MVPA) | *Individual level: a*ge, sex, race/ethnicity, education, annual income, nativity, English language proficiency, length of time in neighborhood | **Neighborhood social cohesion**  OR=1.04 (1.03-1.05)，P <0 .001;  non-Hispanic black or Asian American adults (Chinese, Filipino, and Asian Indians)  *Not significant* |
| (Clarke et al., 2017) | Longitudinal | Baseline N1=584,(Mage=78.5±7.7 )  follow-up  N2=339,(Mage=77.4±7.4),AR≥65 | Tayside, Scotland | community-dwelling older adults | social connectedness(social networks),support networks. | Accelerometers measure physical activity over 7 days. | *Individual level:* health-related quality of Life，psychological factors，comorbidities，anxiety and depression，deprivation Status | *Univariate analysis*  Both provide mixed results  *Multiple regression mode*  **Satisfying friendship network**  β=9,040 , P=0.049;  The others are *not significant* |
| (Fang et al., 2017) | Cross-sectional | N=609,(Mage=60.87±6.91, AR≥18) | Shanghai, China | diagnosed with coronary heart disease | Social participation，social network，social support，social trust，sense of belonging(satisfaction) | Physical activity | *Individual level:* marital status,education level | **Social participation** β=0.25 , P<0.001;  **social network** β=0.10 , P<0.05;  social support β=0.04 , *Not significant*;  **social trust** β=0.18 , P<0.001;  **sense of belonging(satisfaction)** β=0.11 , P<0.01 |
| (Xue & Cheng, 2017) | Cross-sectional | N=28916,(Mage=45.7, AR≥16)Nationally representative data | China | Na | Social Trust,Social Relationship (network),CCP Membership (Participation) | Physical activity | *Individual level:*age, gender, area of living (urban/rural), education, marital status, family size, income, job status | **Social Trust**  OR=1.113(1.044-1.186) ,*p*  < 0.001  **Social Relationship (network)** OR =1.164(1.079-1.257), *p*  < 0.001  **CCP Membership (Participation)** OR =1.709(1.535-1.903), *p*  < 0.001 |
| (Kamimura et al., 2017) | Cross-sectional | N=374,(Mage=46.01±14.28, AR≥18) | America | Uninsured primary care patients with or without hypertension and/or diabetes(socioeconomically disadvantaged populations) | Social capital(social network,control,support,social cohesion) | Physical activity | *Individual level:* age, gender, race/ethnicity, education level, employment status, marital status, country of origin, years in the United States, patient of the clinic for ≥2 years，Health Conditions | **Membership (social network)** OR = 1.68 (1.16-2.46),  *p*  < 0.01  Influence (control) OR = 0.71 (0.50-1.03) *Not significant*  **Reinforcement** (support ) OR = 1.65 (1.11-2.46) *p*  < 0.01  shared emotion ( Social cohesion) OR = 0.77 (0.53-1.13) *Not significant* |
| (Kim et al., 2017) | Cross-sectional | N=8800,(Mage=70.1, AR≥19) | South Korea | in communities with high mortality in Korea | Social participation (formal and informal);Generalized trust; perception of community problems(safety);perceived control | Physical activity | *Individual level: g*ender, age, marital status, educational level, occupation, food security (proxy for socioeconomic status), administrative unit  , self-rated health | **Social participation** *formal and informa****l***  AOR=1.25，(1.10-1.41)  **Generalized trust**  AOR=1.36，(1.19-1.54）  **Perceived control**  *both the community and individual levels*  AOR=1.31,(1.17–1.48)  Perception of community problems AOR=0.99,(0.87-1.13) |
| (Kikuchi et al., 2017) | Cross-sectional | N=1,146,(Mage=70.1, AR=65-74) | Tokyo, Japan | Japanese older adults (not engaged in full- or part-time work) | Social particpation | Physical activity ( MVPA) | *Individual level:* age, gender, educational attainment (years of education), living arrangement (living with others or alone), body mass index (BMI), and physical limitation status | **Social particpation**  *Male* OR=2.10,(1.44-3.06), *p* < 0.001;  *female* OR=1.93, (1.39-2.68),*p*  < 0.001 |
| (Dlugonski et al., 2017) | Cross-sectional | N=86, (Mage= 39.2±9.6, AR=18-64) Female | Greenville，  America | Female participants only (mothers) | Social cohesion and trust, informal social control, social support | Objectively measured physical activity (pedometer) | *Individual level:* age | social cohesion and trust *Not significant* informal social control *Not significant*  **social support** *for Minutes* β=0.26 , p=0.04  *for Step Counts* β=0.21 , p=0.1 *Not significant* |
| (Yuma-Guerrero et al., 2017) | Cross-sectional | N=2750 (Mage=24;AR≥15) Female | California，  America | Female participants only (mothers) | Social cohesion, perceived neighborhood safety | Physical activity | *Individual level:* race/ethnicity, age, obesity status, depression, marital status, number of children under 10 in the | **Social cohesion,**  β=0.103 , p=0.044  Perceived Neighborhood Safety  β=-0.063, p=0.062 *Not significant* |
| (Bartshe et al., 2018) | Cross-sectional | N=410 (Mage=24;AR=18 ~ 61) | Las Vegas，  America | Mostly Caucasian and college students | Social Capital(7 items social cohesion;2 items trust; 16 items social participation) | Physical activity (recommendation of 150 min per week) | *Individual level:* age, gender, and owned a vehicle | **Social Capital**：OR=1.25, p=0.04. |
| (Dwyeret al., 2018) | Cross-sectional | N=1405 adult, (AR=18 ~ 60), (nationally survey) | America | Mostly Non-HispanicCaucasian | 2 items Neighborhood social capital. (reciprocity)  1 item Crime, | Physical activity (Meeting MVPA Guidelines) | *Individual level:* age, gender,race  /ethnicity,education, residential tenure, and self-rated health status. | **Adjusted for Neighborhood Social Capital**: OR= 1.23 (1.03-1.47), p<0.05; Adjusted for Crime: OR=0.96(0.82-1.12) p=0.58; *Not significant* |
| (Flórezet al., 2018) | Cross-sectional | N=799 (Mage=55.9) | Pittsburgh, America | African American,  low-incomeneighborhoods | Social networks (size, diversity) social support | Physical activity (Measuring MVPA while using a wearable accelerometer) | *Individual level:* Age, education,  employment status,  marital status, physical limitations, *Neighborhood level:* income,  neighborhood of residence | Adjusted for social network size: *men:* IRR=1.00, p>0.05; *Not significant*  *women:* IRR=1.00, p>0.05;*Not significant*  **Adjusted for social network diversity**: men: IRR=5.2, p>0.05*Not significant*;women:β=-2.02, IRR=0.13, p<0.01;  social support**:***Not significant* |
| (Fu et al., 2018) | Cross-sectional | N=1210 (Mage=74.3, AR>65) | Wuhan  China | Na | Bonding social capital, bridging  social capital (social network and social cohesion) | Physical inactivity | *Individual level:* Age, gender, marital  status, living condition, education, personal income | **Midlevel bonding social capital:** OR=0.54 (0.37-0.97); **High level bonding social capital:** OR=0.39 (0.21-0.65); **Midlevel bridging social capital:** OR=0.40 (0.22-0.76); **High level bridging social capital**: OR=0.27 (0.15-0.49) |
| (Haynie et al., 2018) | Cross-sectional | N=132 | Pennsylvania, America | Inmates | Social networks (Peer social integration) | Physical activity (exercise frequency) | *Individual level:* Race-ethnicity, religious identity,， smoking status, depression, and exercise intensity | Outdegree *Initiate social behavior* b = -0.142, p > 0.1,*Not significant*  **Indegree** *High social acceptance* b = 0.234, p < 0.05  Match *homophily* (b = 0.154,p>0.1,*Not significant* |
| (Ho et al., 2018) | Cross-sectional | N=738 (Mage=71.9 AR= 62-91) | America | Older adults | Social networks (network size, network proportion friends)  Social engagement (socializing, community involvement) | Physical activity (accelerometer-measured PA) | *Individual level:* Age, gender, education, marital status, employment status, networth, Comorbidities  functional status, cognitive and mental status, self-rated health status | **Adjusted for social network: network size**:β=4.77 (0.18-9.35), p<0.05; **network proportion friends:** β=35.81 (7.97-63.66), p<0.05; **Adjusted for social engagement**: socializing: β=8.73(1-16.46), p<0.05; community involvement: β=3.33(-0.35-7.02), p>0.05, *Not significant* |
| (Josey & Moore, 2018) | Longitudinal | N=2696 (AR= 25-75) | Montreal Metropolitan Canada | Urban-Dwelling Adults | Social networks (exercising alters), network capital, social participation,  generalized trust, | Physical inactivity (calculate the energy costs of activities as the MET) | *Individual level:* Age, gender, marital status, household language, socioeconomic status | **Exercising alters**(Exercise's social connections): OR=0.74(0.66-0.84), p<0.001; **network capital**: OR=0.86(0.76-0.97), p<0.05; **high social participation**: OR=0.67 (0.46-0.98), p<0.05; generalized trust:OR=1.03 (0.82-1.29), p>0.05,*Not significant* |
| (Lightner et al., 2018) | Longitudinal, prospective | N1=3617 (Mage=53.6, AR= 25-95), N5=1427nationally representative sample | America | Initially oversampled Blacks and older adults (60+ years) | Social integration (social network) | Physical activity | *Individual level:* Age, gender, education, race, marital status | **Social integration**: β=0.12 p<0.05 |
| (Marquez et al., 2018) | Randomized controlled trial | N=102, Female (Mage=40, AR= 18-65) | America | Latinas | Social networks (size, density, transitivity, components),network support | Physical activity (Recall Interview and accelerometers.MVPA) | *Individual level:* Age, income, education, employ  ment status, nativity status, English language use, marital status | **Social networks** *Self-reported MVPA:*  **size** β=9.20,p≤0.05  density, transitivity, components *Not significant*  *Objective MVPA: all Not significant*  **Network support**  *Self-reported MVPA:*  *Instrumental support* Chores β=55.98, p≤0.05; Exercise with you β=43.11, p≤0.05  *Emotional support* Complimentsβ=37.67, p≤0.05;  *Objective MVPA Instrumental support*  Exercise with you β=32.09, p≤0.05;  *others Not significant* |
| (Rodrigues et al., 2018) | Cross-sectional | N=3667 (Mage=41) | Belo Horizonte,  Brazil | Na | Social cohesion, collective efficacy | Leisure-time physical activity | *Individual level:* age, sex, marital  status  *Neighborhood level:* educational attainment  socioeconomic position, length of residence in the neighborhood | **Social cohesion:** PR=1.56 (1.13-2.16), p<0.05; Collective efficacy: PR=0.87 (0.64-1.19), p>0.05, *Not significant* |
| (Seino et al., 2018) | Cross-sectional | N=8592 (AR= 65-84) | Tokyo, Japan | Na | Relationships (NRs), (individual NRs, community NRs), social networks | Physical activity  moderate-to-vigorous physical activity  (MVPA); vigorous physical activity (VPA);  moderate physical activity (MPA); walking time (WT); | *Individual level:* age, living situation,  duration of residence in the neighborhood,  education, alcohol drinking and tobacco smoking statuses, body mass, number of chronic diseases  mobility limitation,  self-rated health,  depressive mood,  employment, and  social activity | **Individual NRs (men):**  MVPA, VPA, MPA, WT all  P < 0.041;  **Community NRs (men):** MVPA: β=2.1 (0.7-3.4), P<0.01; MPA: β=4.2 (-2.4-10.8), p=0.216 *Not significant*; VPA: β=8.6 (2.7-14.4), p<0.001; WT: β=11.6 (2.2-20.9), p<0.05.  **Individual NRs** (women):  **visiting each other:**NR, MVPA, VPA, WTallP < 0.009;  Community NRs (women)**:***all Not significant* |
| (Vancampfort et al., 2018) | Cross-sectional | N = 915 (Mage = 72.8, AR>65) | China, Ghana, India, Mexico, Russia, South Africa | Older adults with depression | Social cohesion | Low physical activity levels (not meeting 150 minutes  of moderate PA per week) | *Individual level:* health behaviors, mental health, physical health | **Social cohesion:** OR=0.96 (0.94-0.98), p<0.001 |
| (Yamamoto & Hyerim, 2018) | Cross-sectional | N=530 (Mage= 42.49) | Chicago，America | Online panel of participants who lived in the city of Chicago | Social cohesion，social networks(anonymity, weak-tie communication) | Physical activity (frequency) | *Individual level:* age, gender, education, income, race, marital, number of children, length of residence, home ownership | Social cohesion: β=0.07, *Not significant;*  **social networks**  Anonymity β = −0.19，p <0.05；  Weak-tie communication β=0.06, Not significant; |
| (Yildizer et al., 2018) | Cross-sectional | N=1235 (AR= 14-18) | Turkey | Urban public schools | Social capital (neighborhood trust, informal social control, teacher–student interpersonal trust, student interpersonal trust, students’ collaboration in school/ collective efficacy) | Physical activity (≥60 min/day in moderate, vigorous, and walking physical activity) | Individual level:  gender, age, socioeconomic status, health behaviors, mental health, physical health | Neighborhood trust: OR=0.98 (0.75-1.27), *Not significant;*  informal social control**:** OR=0.95 (0.71-1.27), *Not significant*; **teacher–student interpersonal trust:** OR=0.74 (0.56-0.97), p<0.05; student interpersonal trust: OR=1.01 (0.75-1.35), *Not significant*  students’ collaboration in school: OR=0.79 (0.60-1.03), *Not significant* |
| (Yildizer et al., 2019) | Cross-sectional | N=520(AR= 14-18) | Eskişehir, Turkey | high school students | Family support, Neighborhood trust, school social capital (informal social control, trust between teachers and students, interpersonal trust among students, collaboration/ collective efficacy) | Overall physical activity participation (OPAP)& moderate-to-vigorous physical activity participation (MVPAP) | Individual level:  self-rated health, body mass index, BMI, self-perceived socioeconomic status, SES | OPAP: Total all *Not significant*  **Family Support:** *for female* OR = 2.53，[1.33-4.79]，p <0.05.  *male* OR = 0.50，[ 0.22-1.11], *Not significant*  **Student Interpersonal Trust**  *for female* OR = 0.49，[0.24-0.96]，p <0.05  *male* OR=0.97，[0.51-1.85], *Not significant*  MVPAP : Total all *Not significant*  **Student Interpersonal Trust**  *for female* OR = 0.35，[0.14-0.84]，p <0.05  *male* OR = 0.94，[0.56-1.60], *Not significant* |
| (Chen et al., 2019) | Cross-sectional | N=600 (AR=15-69) | China | Na | 24 items social capital (social network, social support, social participation, control over life, feeling about the community/ satisfaction) | Physical activity | Individual level:  age, gender, educational attainment, health literacy | **Social network:** β=1.572, p<0.001; **social support** β=0.768, p<0.001;  **social participation:** β=0.758, p<0.001; **control over life**: β=0.933, p<0.001; **feeling about the community**: β=0.868, p<0.001 |
| (Jun & Park, 2019) | Cross-sectional (nationwide survey) | N= 228558 (Mage=53.15) | Korea | Na | Trust, social network, reciprocity, | Physical activity | Individual level:  age, gender, education, working status, length of residence, job type, income, household size, subjective health status  *Community Level:* population density (logged)，land-use mix, area of parks per person, number of sports facilities per 1000 people, percentage of area for housing complexes, fiscal self-reliance ratio | **Trust:** β=0.124, p<0.01; **social network:** β=0.091, p<0.01; **reciprocity:** β=0.180, p<0.01 |
| (Quinn, 2019) | Cross-sectional (national survey) | N=23006 (Mage=47.2) | America | Na | Neighborhood cohesion, (help availability/ reciprocity, trust, network, responsibility) | Physical activity (aerobic, strength, aerobic and strength) | Individual level:  age, sex, race/ethnicity, family-income-to-poverty ratio, neighborhood tenure, education, US nativity, and English language proficiency | **Neighborhood cohesion:** *Guideline Min/Wk* β=45.0, p<0.001; *Meeting Aerobic Guidelines* OR = 1.22 (1.13–1.31); *Meeting Strength Guidelines* OR = 1.13 (1.04–1.23); *Meet both criteria* OR = 1.14 (1.05–1.25)  **help availability:** β=40.9, p<0.001; **trust:** β=35.0, p=0.001; **responsibility:** β=48.3, p<0.001; **network:** β=40.1 ,p<0.001 |
| (Wang et al., 2019) | longitudinal (national survey) | N=24620 (AR>60) | China | Na | Neighborhood social reciprocity | Physical activity (frequencies) | Individual level:  gender, age, educational attainment, marital status, household size, annual household income, rural/urban residence, cigarette use, alcohol use, self-reported physical health status, functional ability, medical insurance | **Neighborhood social reciprocity**: β = 0.293, p<0.01 |
| (Davis et al., 2020) | Cross-sectional | N=1193 (AR=65-74) | Tirana, Albania  Manizales, Colombia  Natal, Brazil | Older adults from three middle-income countries | Social networks (social ties), social participation (religious activities), perceived safety | meeting WHO PA guidelines of 150 weekly walking minutes yes/no (using the Mobility Assessment Tool for Walking) | *Individual level:*  diabetes, depression, age, sex, tertile of education (by site), income sufficiency, alcohol consumption, partner ties, child ties, friend ties, usual source of care, frequent visits to the doctor, participation in religious activities, perceived safety | **Social networks**  High partner ties (vs. low) OR = 1.38， [1.04，1.83]  High friend ties (vs. low) OR = 1.39， [1.05，1.83]  High child ties (vs. low) OR = 0.97， [0.72，1.31] *Not significant*  social participation religious activities (vs. none) OR = 1.13， [0.73，1.76], *Not significant*  High perceived safety (vs. low to moderate) OR = 0.89， [0.66，1.21] *Not significant* |
| (Fingerman et al., 2020) | Cross-sectional | N=313 (AR>65) | Texas, America | Na | Social networks (social ties) | Physical activity (measured by actical accelerometers) | *Individual level:*  age, gender, marital status, education, racial/ethnic status, and global physical health. | **Diversity of Social Ties:** B = 985.03，SE = 104.35，p < 0.001  Close Ties B =-1149.63 *Not significant*  **Peripheral Ties** B = 2712.20, SE = 251.09, p < 0.001 |
| (Heredia et al., 2020) | Cross-sectional | N=1009 (Mage=49) | Texas, America | African American church members | Social participation, social support, social cohesion, social norms | Physical activity (task (MET) minutes/week for PA was calculated) | *Individual-Level:* age, gender, education level, employment status, perception of risk for cancer, worry about getting cancer, perceived stress, depression, loneliness, financial strain.  *Neighborhood-Level:* presence of sidewalks, presence of parks/trails, neighborhood safety for physical activity, neighborhood problems. | Social participation OR =1.01 [0.88，1.40] *Not significant*  social support OR =1.01[0.99，1.04] *Not significant*  **social cohesion** OR = 1.05 [1.00, 1.11] , p<0.05  **social norms** *for FV intake* OR =1.64[1.15，2.34], p<0.01; *for PA* OR =1.60 [1.02, 2.51], p<0.05 |
| (Kim et al., 2020) | Cross-sectional (national survey) | N = 6,412 (Mage=70±7) | America | Na | Neighborhood social cohesion | Leisure time physical activity (light-to-moderate and vigorous) | *Individual-Level:* age, gender, length of residence, physical condition | **Neighborhood social cohesion** *light-to-moderate LTPA* β =0.05, p <0.001；  *vigorous LTPA* β = 0.03, p = 0.015 |
| (Ward et al., 2020) | Cross-sectional | N=10540 (AR>55) | Ireland | Adults Aged 55 and Older in Ireland | Community Participation (social participation), perceived safety, social network | Physical activity | *Individual-Level:* gender, age, marital status, household composition, education, employment status, material deprivation, location (urban or rural), driving status. body mass index, smoking status, self-rated health, presence of limiting illness, mobility difficulties, and doctor-diagnosed mental illness. | **Social participation**  *weekly* β = 0.304, p ＜0.001;*Monthly* β = 0.124, *Not significant*;  **social network** β = 0.043, p ＜0.05 :  **perceived safety**  *Feels safe out during the day*  *Female not safe* β = −0.220 *Not significant*;  *Male safe* β = 0.285 p < 0.001  *Male not safe* β = −0.461 p < 0.05  *Feels safe out at night* β =  0.001 *Not significant* |
| (Aliyas, 2020) | Cross-sectional | N=1828 (AR=18-72) | south of Iran | Urban Adult Population | Reciprocity, trust, social network | Physical activity (vigorous, moderate) | *Individual-Level:* age, gender, marital status, education level, occupation, residential stability, type of residence, health-related variables | **Reciprocity:**  *female:* OR=2.12, [1.73-2.58], p < 0.01  *male:* OR=1.56, [1.29-1.90], p < 0.01  **Trust:**  *female:* OR=1.43, [1.24-1.64], p < 0.01  *male:* OR=1.31, [1.14-1.50], p < 0.01  **social network:**  *female:* OR=1.44, [1.25-1.66], p < 0.01  *male:* OR=1.12, [0.96-1.31], *Not significant* |
| (Dlugonski et al., 2020) | Randomized controlled trial | N=74 (Mage=39.2±9.6) | America | Mothers | Collective efficacy | Physical activity (using pedometers) | *Individual-Level:* age, education, income, employment status, marital status, body mass index (BMI). | **Collective efficacy**: *active minutes*: p = 0.05  Number of steps: p = 0.049 |
| (Thanawala et al., 2020) | Cross-sectional | N=689 (Mage=59±9 AR=43-85) | America | South Asians | Social networks | Physical activity (MVPA) | *Individual-Level:* age, gender, education, income, marital status, and study site (San Francisco or Chicago), cultural beliefs, self-rated health, network size | **Number of Exercise Partners**: For Males: p < 0.01; For Females: No significant  **Number of Exercising Alters**: *For Males:* p < 0.01; *For Females: No significant*  **Non-Spouse Exercise Partners:** *For Males:* p < 0.01; *For Females: No significant*  **Spouse exercise partner：**For Males: No significant; For Females: p < 0.05 |
| (Liang et al., 2020) | Cross-sectional (national survey) | N=3719 (Mage=68.83 AR≥60) | China | elderly individuals residing in rural China | Structural Social Capital (social network, social participation), Cognitive Social Capital (trust, reciprocity, mutual assistance,) | Physical activity (frequency of exercise per week) | *Individual-Level:* sex, age, education level, marital status, social status, positive attitude, self-rated health, others-rated health. | **Structural Social Capital:** β =0.385，P <0.001  **Cognitive Social Capital:** β =0.249，P <0.001 |
| (Boen et al., 2020) | Cross-sectional and longitudinal | N1=959, N2=409 , AR≥55 | Belgium | Participants were members of OKRA | Safety, social engagement, volunteer engagement, connectedness with OKRA, connectedness with community, connectedness with age peers (social networks) | Physical activity (calculate MET-values) | Individual-Level: age, gender | Cross-Sectional Analysis  **Safety** β =1.34，P <0.05  **social engagement**, β =0.64，P <0.05; other all No significant;  Longitudinal Analysis  all No significant; |
| (Zhao et al., 2020) | Cross-sectional | N=520 Female (Mage=61.36 ±6.72) | Shanghai, China | Chinese breast cancer survivors involved in community-based cancer rehabilitation activities. | Cognitive Social Capital (Control over Life,  feeling about the community/ satisfied), Structural Social Capital (social participation  social network  social support) | Physical activity | *Individual-Level:* age, body mass index (BMI), education level, marital status, personal monthly income, duration of disease | **social participation** β =0.054，P <0.001;  **social network** β =0.054，P <0.001;  **social support** β =0.043，P <0.001;  **Control over Life** β =0.072，P <0.001;  **feeling about the community** β=0.058, P <0.001; |
| (Cho et al., 2021) | Prospective Cohort | N=6799 (AR=30-64) | South Korea | Na | Social networks density, closed triads (emotional intimacy) | Self-reported physical activity and accelerometer-assessed (MVPA) | *Individual-Level:* age, network size, socioeconomic status, disease history, obesity, lifestyle factors | Self-reported physical activity  **Network Density:**  *In females* β = -528.6, p = 0.0002  In males β = -346.7, p = 0.2221 (Not significant)  **Closed Triads:**  *In females* β = -641.5, p < 0.0001  In males β = -542.6, p = 0.0551 (Not significant)  MVPA**:** (*Not significant)*  *In males:* density β=229.7, p=0.3193;  closed triad β=109.21, p= 0.6333 |
| (Feng et al., 2021) | Cross-sectional | N= 13367 (Mage=63.2) | China | Middle-Aged and Older Chinese People | Social cohesion | Physical activity (WHO-recommended thresholds) | *Individual-Level:* age, gender, marital status, area of residence, educational level, income | Social cohesion:  OR = 1.058; [0.975–1.147] *Not significant* |
| (Mieziene et al., 2021) | Cross-sectional | N=1285 students (Mage=16.14±1.22, AR=14-18) | Lithuania | Senior high school student | Family social capital (family support); Neighborhood social capital (social support, social trust, informal social control); School social capital(cohesion)social network, social participation | Physical activity (MVPA) | *Individual-Level:* age, gender, parental education, socioeconomic status, place of living (urban vs. rural), and other sociodemographic factors | **Social Participation:** OR= 1.03 [1.01-1.05];  Family Social Capital: OR=1.04 [0.98–1.09]  *Not significant*  Neighborhood social capital: OR=0.98 [0.86–1.12], *Not significant*  School social capital: OR=0.96 [0.90–1.01], *Not significant*  Social network: OR=1.03 [0.99–1.07], *Not significant* |
| (Montgomery et al., 2021) | Cross-sectional | N=529 (Mage=14.38±0.32) | Northern Ireland | Na | Social networks | Physical activity | *Individual-Level:* age, gender, and social network characteristics like popularity (in-degree) and sociability (out-degree). | **Social networks:**  Friend group  in female β =0.25, p = 0.14, *Not significant*  in male β =0.46, p = 0.007；  Best friend’s  in female β =0.21, p = 0.03；  in male β =0.21, p = 0.09, *Not significant* |
| (Nemoto et al., 2021) | Longitudinal | N=3833 (AR≥65) | Yamanashi, Japan | Japanese older adults | Social engagement | Physical activity (MET),vigorous-intensity physical activity (VPA),  moderate-intensity physical activity (MPA) | *Individual-Level:* sex, age, educational attainment, employment status, household status, marital status, self-rated health, medical history, mental health | **Consistent Engagement:**  *Total Physical Activity:* β = 2.3, [0.9, 3.7], p < 0.01  *VPA:* β = 6.2, [-0.7, 13.1], p =0.11, *Not significant*  *MPA:* β = 17.0, [9.9, 24.1], p < 0.01  Leaving the Group:  Total Physical Activity: β = 1.5, [-0.2, 3.2], p = 0.12, *Not significant*;  *VPA:* β = 5.9, [-2.9, 14.6], p =0.46, *Not significant*；  *MPA*: β = 11.3, [2.4, 20.3], p < 0.01;  New Engagement:  *Total Physical Activity*: β = 3.3, [0.9, 5.6], p < 0.01  *VPA:* β = 8.7, [-3.1, 20.7], p =0.31, *Not significant*  *MPA*: β = 13.7, [1.5, 26.0], p = 0.02 |
| (Pérez et al., 2021) | Longitudinal | N=98, (Mage=82.7) | Barcelona, Spain. | during the COVID-19 lockdown | Maintenance social network | Physical activity | Individual-Level:  gender, age, education level, living situation, social relationships, depress symptoms, fatigue, other health-related factors | **Maintenance social network:**  OR=5.07, [1.60, 16.08], p=0.006 |
| (Yamashita et al., 2021) | Randomized controlled trial | N=39 female (AR≥65) | Kumamoto, Japan | Na | Social network incentives | Physical activity (pedometers) | Individual-Level:  age, body weight, body height, body mass index (BMI), percentage of body fat | **Social network incentives:** interaction of time and groups  F (1, 37) =6.24，p  = 0.017  *before and after intervention* F (2, 36) =5.41，p  = 0.006 |
| (Sasaki et al., 2021) | Cross-sectional | N=999 community-dwelling residents. (AR=65-90) | northern Japanese | Older People during the COVID-19 Pandemic | Social participation | Physical activity (increasing or maintaining MVPA) | Individual-Level:  age, sex, body mass index (BMI), smoking status, alcohol consumption, self-reported health status, living arrangements | Social participation:  *in female* OR=1.67, [1.13–2.45], P=0.0094  *in male* OR=1.48, [0.74–3.00], p=0.27, *Not significant* |
| (Herbolsheimer et al., 2021) | Longitudinal observational | N=2286 (Mage=73.8±5.0) | Six European countries (Germany, Italy, the Netherlands, Spain, Sweden, and the United Kingdom) | Participants with lower limb osteoarthritis (LLOA) | Social participation | Physical activity | *Individual-Level:* age, average temperature, Body Mass Index (BMI), self-rated health | **Social Participation:** cross-sectional:  *No LLOA:* β = 0.11, p < 0.01;  *LLOA:* β = 0.15, p < 0.05  longitudinal:  *No LLOA*: β = 0.01, *Not significant*  *LLOA*: β = 0.18, p < 0.10, *Not significant* |
| (Liu & Lachman, 2021) | Randomized controlled trial | N=60 (Mage=65.7) | Northeastern America | Older adults | Social network (Social contact & comparisons using WeChat) | Physical activity (Daily Steps: Tracked using the WeChat application.) | *Individual-Level:* age, gender, education, race, self-reported health | **Social network:** Social contact & comparisons using WeChat β = 1.06, [0.97, 1.18] |
| (Otaki et al., 2022) | Cross-sectional | N=1925 (Mage=74.1±6.1, AR=65-90) | Japan | Community-dwelling older adults in Japan due to COVID-19 | Interfered Social contact (social networks & social participation) | Physical activity (MET-min/week) | *Individual-Level:*  sex, age, BMI, alcohol use, smoking, living alone, number of illnesses | **In-person contact with friends** β=-0.111; P < 0.001  *others Not significant* ;  **social participation** β=-0.163; P < 0.001 |
| (Wang et al., 2022) | Cross-sectional | N=2590 (Mage=40.4,AR=18-75) (a nationally representative sample) | America | Native Hawaiian and Pacific Islanders (NHPI) | Neighborhood social cohesion | Leisure time physical activity | *Individual-Level:* sex, age, multiracial status, income, education, employment status, marital status | **Social cohesion** OR=1.59,(1.19–2.12), p=0.003 |
| (Wieland et al., 2022) | Single-arm, non-randomized, pilot | N=39 (Mage=48) | Southeastern Minnesota, America | Immigrant populations (Hispanic and Somali) | Social network intervention delivered by lay health promoters (HPs) | Physical activity (measured in MET-min/week) | Na | **Social network**  *Increased physical activity* P = 0.02 |
| (Zimmer & McDonough, 2022) | Cross-sectional | N=21494, (AR≥65) (National representation) | Canada | Canadian older adults | social networks (marital/partner status, size, social contact online communication,positive social interaction) social participation, tangible support, affectionate support, emotional/informational support | Physical activity | *Individual-Level:* age, sex, education, BMI, general health, mental health, depression, chronic conditions, pain, injuries, falls, smoking habits, alcohol use, social inequality, retirement status, barriers to social and physical activities | **Social network size** b=0.04, p<0.05  **Social contact frequency** b=2.93, p<0.05  **Social participation** b=7.92, p<0.05  Marital/partner status  b=−5.60; p<0.05  Tangible support  b=−0.08,p<0.05  *others* *Not significant* ; |
| (Ganzar et al., 2022) | Longitudinal | 168 children (AR=8-11) | Central Texas, America | Socioeconomically and ethnically diverse sample of school-age children during the COVID-19 pandemic | Neighborhood social cohesion, informal social control, safe | Moderate-to-vigorous physical activity (MVPA) (using accelerometers) | *Individual-Level:* age, sex, race/ethnicity | **Neighborhood social cohesion**  OR=1.21, (1.04, 1.44), p  < 0.01  informal social control OR=1.06（0.98, 1.17）*Not significant* ;  safe *Not significant* ; |
| (Du & Li, 2022) | Cross-sectional | N=14,595 (AR=24-32) | America | Young adults | Social networks (relationship quality, contact frequency, network size, spatial accessibility) | Moderate-to-vigorous physical activity (≥5 times of activity/week) | *Individual-Level:* gender, ethnicity, income, educational attainment | **Social networks** (relationship quality  , contact frequency, network size, spatial accessibility) provide mixed results |
| (Cheng & Nicklett, 2022) | Longitudinal | N=17974 (AR≥50) (nationally representative) | America | middle aged and elderly people | Neighborhood social cohesion,  neighborhood physical environment(Satisfied with environment) | Physical activity measured in METs (light, moderate, vigorous activities) | *Individual-Level:*  age, gender, race/ethnicity, education, wealth, health-related factors (functional limitations, chronic illnesses, depressive symptoms) | **Neighborhood social cohesion** β=0.24 (0.18, 0.31),p < 0.001.  **neighborhood physical environment**  β=0.11 (0.04, 0.17),p < 0.01. |
| (Crandall et al., 2022) | Longitudinal | N=41,443 older women (Mage=83.2±5.4, AR=65-90) | America | During the COVID-19 Pandemic Among Older Women | Social connectedness (Social network) | Physical activity levels before and during the COVID-19 pandemic | *Individual-Level:* age, race/ethnicity, BMI, physical function level, education | **Decreased physical activity**  OR = 0.91（0.87-0.95），p < 0.001  Increased Physical Activity:OR=0.99 (0.93–1.07), p = 0.87 |
| (Wray et al., 2022) | Cross-sectional | N=73,(Mage=12.04.±0.9) | Missouri, America | middle-school youths | Social integration (social networks) | Physical activity (MVPA) | Na | **Social networks**  *Time with Friends* **β=**0.33 p= 0.02;  *Time with Family*  **β=**0.01, p= 0.94 *Not significant* |
| (Sugiyama et al., 2023) | Cross-sectional | N=3,055 pregnant women, (AR=17-45) | Miyagi Prefecture, Japan | Japanese pregnant women | Emotional support, neighborhood trust, perceived safety, generalized trust | Physical activity (≥ 150 min/week) | *Individual-Level:* age, education level, parity, smoking status, drinking status, marital status, family members, gestational weight gain, pre-pregnancy BMI, household income, morning sickness, employment, depressive symptoms, pregnancy complications, living in temporary housing | *During Pregnancy*  **Emotional support** OR=1.45 (0.95-2.22), p = 0.002；neighborhood trust OR=1.17 (0.94-1.47), p = 0.071；*Not significant;* perceived safety OR=1.03 (0.77-1.37),p = 0.771, *Not significant;* generalized trust OR=1.15 (0.87-1.51); p = 0.800 *Not significant*  *1.5 Years After Delivery*  **Emotional support** OR=1.66 (0.90-3.05), p < 0.001；neighborhood trust OR= 0.99 (0.67-1.45), p = 0.916；*Not significant*  perceived safety OR=1.03 (0.77-1.37); p = 0.771,*Not significant；*  generalized trust OR=1.17 (0.81-1.68), p = 0.547,*Not significant* |
| (Akosile et al., 2023) | Cross-sectional | N=170 (AR≥65) | Nsukka, Enugu State, Nigeria | Nigerian older adults from rural and urban communities | Neighborhood safety | Physical activity level | Na | Neighborhood safety r = 0.04, p = 0.60, *Not significant* |
| (Bartshe et al., 2023) | Cross-sectiona | N=403,(Mage=24.±7.42) | Las Vegas, Southern Nevada, America | college students | Social capital(social cohesion, social participation, trust, informal social contro) | Physical activity (met physical activity recommendations) | Na | **Social capital**  OR = 1.308,(1.067, 1.605), p = 0.01 |
| (Prochnow et al., 2023) | Cross-sectiona | N=250,(Mage=20.25.±1.65) | Southern America | college students | Social networks developed through intramural sports | physical activity (meeting physical activity recommendations.) | Na | **Social networks**  *Percent often Physically*  OR=2.47(1.08,5.62),P=0.03  *Percent improved through intramurals*  OR=2.36(1.83,3.87),P=0.01  average closeness, percent met through intramurals *Not significant* |
| (Kronaizl & Koss, 2023) | Longitudinal | N=4441 families (children assessed at ages 5, 9, and 15 years) | America | adolescent | Neighborhood collective efficacy (Maternal self-reported perceived NCE at age 5) | physical activity at age 15 | *Individual-Level:* maternal age, education, race/ethnicity, household income, pubertal development, television watch time, maternal depression, child's BMI at age 9 | Neighborhood collective efficacy Β = 0.05，p =0.390 |
| (Xie et al., 2024) | Cross-sectional | N=2504,(Mage=15.48.±0.68, AR=14-18) | America | adolescent | Neighborhood social ties (social network) | Physical activity levels | *Individual-Level:* youth gender, age, race/ethnicity, primary caregiver's education level, marital status, household income/poverty ratio, family size | **Neighborhood social ties**  b=0.21, p < 0.01 |

Note. M = Male, F=Female, Mage = Mean Age, AR= Age Range, Na = Not available，MET=Metabolic Equivalent of Task，MVPA=Moderate-to-Vigorous Physical Activity.
